# Does text generation improve learning from expository text? A conceptual replication attempt

**DOI:** 10.1186/s41235-025-00645-2

**Published:** 2025-06-23

**Authors:** Julia Schindler, Tobias Richter

**Affiliations:** 1 Department of Psychology IV, University of Würzburg, Röntgenring 10, 97070 Würzburg, Germany; 2 Department of Education and Psychology/Special Needs Education, Free University of Berlin, Fabeckstraße 35, 14195 Berlin, Germany

**Keywords:** Conceptual replication, Expository texts, Generation effect, Learning, Text generation

## Abstract

The aim of the present study was to test the replicability of the text generation effect for learning with expository texts while systematically varying contextual factors that—based on extant literature—can be assumed to affect the occurrence and magnitude of the text generation effect. Seven experiments were conducted in which participants either read (control condition) or unscrambled sentences (generation condition) in expository texts. The experiments varied systematically on intentionality of learning, learning time constraint, retention interval, and study design. Contrary to expectations, no text generation effect could be found. Instead, some of the experiments even revealed a learning disadvantage for text generation compared to the reading control condition. In only one experiment (Experiment 6) and for just one of the learning measures, learners performed better when they had generated the texts. In sum, the results indicate that a generation effect is most likely to occur when learning is intentional, when learning time is unrestricted, and for immediate testing. The findings suggest that the applications of text generation in educational contexts are rather limited.

**Supplementary Information:**

The online version contains supplementary material available at 10.1186/s41235-025-00645-2.

## Introduction

A common misapprehension is that learning is usually most effective when it is experienced as easy and fluent (Bjork et al., 2013). Conditions increasing the perceived ease of learning such as priming, high encoding fluency, or perceptual fluency often have detrimental effects on long-term memory because they can create an illusion of knowing, which leads learners to terminate learning too early (Bjork et al., 2013). Considerably less intuitive is the finding that under certain conditions learning is more effective when it is intentionally made more difficult instead of easier (Bjork & Bjork, 2011). These specific learning conditions are known as *desirable difficulties* (Bjork, 1994). 

A well-established desirable difficulty is the so-called *generation effect*: Learning can be made more effective and long-lasting when the to-be-learned information is generated by the learners instead of being passively received (e.g., McDaniel et al., 1988; Slamecka & Graf, 1978). The generation effect has been replicated repeatedly in laboratory studies with word pair lists as learning material. In the original paradigm, learners either read a word pair consisting of a context and an associated target word (e.g., winter–SNOW), or a fragmented target word must be completed (generated) by the learners (winter–S_ _ _) (McDaniel et al., 1988; Slemacka & Graf, 1978). These word pair learning studies consistently showed that generated target words are better recalled and recognized than read words (see the meta-analyses by Bertsch et al., 2007 and McCurdy et al., 2020). 

### The genre-by-generation task interaction

The generation effect has also been demonstrated with texts as learning material (e.g., Einstein et al., 1990; McDaniel et al., 1986, 1994, 2002; see also the meta-analysis by Schindler & Richter, 2023), which suggests that text generation might be applicable in educational contexts such as school or university studies. According to Schindler and Richter (2023, p. 44), text generation compared to reading “includes all activities that involve the creation of the text material (or parts of it) such as letter completion in a fragmented text (e.g., *s_me lett_rs ar_ mis_in_ in t_is se_t_nce*) or reordering scrambled sentences (e.g., Sentence 1 in Position 4, Sentence 6 in Position 1, Sentence 2 in Position 3)”. It can thus be distinguished from text elaboration strategies that involve reading and an additional task such as answering questions about the text.

Expository texts play an important role in formal and informal learning because one of their primary purposes is to convey new information in the form of new concepts, arguments, and explanations (Hall-Kenyon & Black, 2010; Mar et al., 2021). Extant research on text generation indicates that the task of sentence unscrambling is especially beneficial for learning with expository texts (e.g., Einstein et al., 1990; McDaniel et al., 1986; Schindler & Richter, 2023), whereas filling in letters is especially beneficial for learning with narratives (e.g., Einstein et al., 1990; McDaniel et al.,) such as fairy tales or stories primarily written for entertainment (Mar et al., 2021).

This genre-by-generation task interaction can be explained by the material appropriate processing framework by McDaniel et al., (1986; McDaniel & Einstein, 1989) and the more comprehensive contextual framework by McDaniel and Butler (2011; see also Einstein et al., 1990; McDaniel & Einstein, 1989, 2005). Both frameworks assume that generation benefits learning only when the generation task stimulates cognitive processes that have not already been stimulated by the text or by the learners (see also Einstein & Hunt, 1980; Hunt & Einstein, 1981). According to the material appropriate processing framework, two types of processing are essential for successful text comprehension and consequentially for learning: (1) item-specific processing, that is, the processing of individual items (idea units or propositions) and (2) relational processing, that is, organizing the individual items and establishing (e.g., conceptual, chronological, causal) relations between them (integration). Learning should thus be effective to the extent that both types of processing are stimulated.

McDaniel et al., (1986; McDaniel & Einstein, 1989) further assumed that narratives and expository texts stimulate these processes to a different extent. Narratives usually follow a familiar story schema (Rumelhart, 1975), which specifically fosters the establishment of relations between propositions. At the same time, they are assumed to stimulate item-specific processing to a lesser extent. Consequentially, generation would only enhance learning with narratives when the generation task complementarily stimulates item-specific processing. Such a task would be, for example, letter completion because the blanks within the words draws the readers’ attention to individual words and idea units (McDaniel & Einstein, 1989; McDaniel et al., 1986). In contrast, tasks that stimulate relational processing should be redundant in learning with narratives and are thus not expected to benefit learning beyond reading the text. One such task is sentence unscrambling which requires organization and integration of sentence content to sort them (McDaniel & Einstein, 1989; McDaniel et al., 1986).

Learners usually have less well-organized schemas for expository texts (McDaniel & Einstein, 1989). Instead, they draw the learners’ attention to individual items or propositions such as new words or concepts and to a lesser extent to the relations between them. Hence, learning can only be improved by a task which complementarily stimulates relational processing such as sentence unscrambling (McDaniel & Einstein, 1989; McDaniel et al., 1986).

### Heterogenous findings and moderating factors

The beneficial effect of sentence unscrambling in expository texts has often been replicated in laboratory studies (Einstein et al., 1984, Experiment 1, 1990, Experiment 2; McDaniel, 1984; McDaniel & Kerwin, 1987; McDaniel et al., 1986, Experiment 2) and was also supported by Schindler and Richter’s (2023) meta-analysis showing a moderate text generation effect for sentence unscrambling, with a substantial effect of Hedges’ *g* = 0.77 when only expository texts were considered.

The overall positive effect of text generation notwithstanding, a considerable number of studies have reported inconsistent findings. Sentence unscrambling has been found to unexpectedly enhance recall for narratives in some studies (Einstein et al., 1990, Experiment 2; McDaniel et al., 1994, Experiments 1–3; Schindler et al., 2024) but failed to increase learning beyond the reading of expository texts in others (McDaniel et al., 2002, Experiment 1B; Schindler et al., 2024, preregistered analyses in the English sample, Thomas & McDaniel, 2007, Experiment 1). These conflicting findings suggest additional contextual factors might moderate the occurrence and magnitude of the text generation effect. The following sections discuss some potential moderators.

#### Intentionality

Most studies of Einstein, McDaniel and colleagues, who first reported the genre-by-generation task interaction, used incidental learning paradigms to test the text generation effect (Einstein et al., 1984, 1990; McDaniel et al., 1986, 1994, 2000, 2002). In these studies, learners were naïve to the upcoming learning test and thus had not actively prepared for it. In academic settings though, learning is often intentional. Learners usually know when a learning test or an exam is scheduled and thus intentionally prepare for it. To be of use in educational settings, studies would need to show evidence of sentence unscrambling as also beneficial in intentional learning contexts. Two meta-analyses by Bertsch et al. (2007) and McCurdy et al. (2020) on the generation of less complex learning material (such as word pairs, sentences, and numbers) reported significantly larger effect sizes for incidental (Cohen’s *d* = 0.65 and *d* = 1.03) than for intentional learning (*d* = 0.32 and *d* = 0.61), suggesting that learners engage in the necessary cognitive processes when preparing for a test, which might render generation partly redundant. However, the meta-analysis by Schindler and Richter (2023) reported mixed findings on intentionality as a moderator of the generation effect when learning with texts. Their analysis of single moderators indicated no significant differences between incidental and intentional learning settings. Their meta-regression analysis, which includes multiple moderators, even suggests significantly smaller effect sizes for incidental than for intentional learning (full regression model). This finding contradicts the meta-analytic findings by Bertsch et al. (2007) and McCurdy et al. (2020) and suggests that learners who expect a learning test might take the task more seriously (Schindler & Richter, 2023).

#### Retention interval

Another possible moderator is the interval between the learning and the test. A successful learning method should benefit not only short-term but also long-term learning. Moreover, desirable difficulties are assumed to specifically benefit long-term learning (Bjork & Bjork, 2011), an assumption that was corroborated in the meta-analyses by Bertsch et al. (2007) and McCurdy et al. (2020) who found the largest effect sizes for generation at the longest retention intervals (more than a day, *d* = 0.64 and *d* = 1.34). Schindler and Richter (2023), in contrast, found no significant differences in effect sizes depending on retention interval. The longest retention interval (1–14 days) was (descriptively) even associated with the smallest effect size (*g* = 0.33). Thus, the role of the retention interval in generating texts still needs to be further investigated.

#### Learning time constraint

Given that sentence unscrambling reduces reading flow, the greater processing time needed compared to reading is not surprising (e.g., McDaniel et al., 1986; Schindler et al., 2024). This difference raises the question whether the generation effect might just be a time-on-task effect. In other words, the learning benefit might not be attributable to beneficial cognitive learning processes but simply to the amount of time spent studying. Bertsch et al.’s (2007) and McCurdy et al.’s (2020) meta-analyses neither considered the moderator learning time restriction (limited vs. unlimited) nor whether the learning time was recorded and statistically controlled in the analyzed studies. Schindler and Richter (2023) reported generation effects for studies with (*g* = 0.45) and without learning time constraints (*g* = 0.37), with no statistically significant differences in effect sizes between both moderator levels. However, they did not consider whether learning time (when unconstrained) was statistically controlled in the studies. Comparing studies with restricted learning time and studies with unrestricted but controlled learning time could clarify whether a pre-set learning time leads to different processing methods (e.g., more superficial or heuristic processing) compared to no time restriction (which allows deep and presumably more time-intense processing).

#### Type of learning test

All three meta-analyses reported the generation effect to occur across all investigated types of learning tests. Bertsch et al. (2007) and McCurdy et al. (2020) reported smallest effect sizes for free recall (*d* = 0.32 and *d* = 0.78) and largest effect sizes for cued recall (Bertsch et al., 2007, *d* = 0.55) or recognition tasks (McCurdy et al., 2020, *d* = 1.09). In contrast, in their meta-analysis on the text generation effect, Schindler and Richter (2023) found the largest effect sizes for learning tests using free recall (*g* = 0.60) compared to cued recall (*g* = 0.27) and no generation effect for studies based on other tasks such as single choice or verification (this last category however contained only five effect sizes). Given that sentence unscrambling should stimulate the construction of relations between the sentences (such as coherence relations between propositions and sentences), the generation effect should occur more prominently for tasks that specifically encourage these processes (such as inferential questions) instead of tasks asking for item-specific information (such as text-based questions).

#### Study design

The extant research has shown that study design might affect generation effect size in favor of within-subjects designs, which generated effect sizes about twice as large as between-subject designs (Bertsch et al., 2007; within: *d* = 0.50, between: *d* = 0.28) or at least significantly larger (McCurdy et al., 2020; within: *d* = 1.08, between: *d* = 0.79) for less complex learning material. However, the meta-analysis by Schindler and Richter (2023) found no impact of study design on the occurrence or magnitude of the text generation effect. Given the comparable findings by Bertsch et al. and McCurdy et al. though, which were based on significantly more studies than the study by Schindler and Richter, study design cannot be ruled out as a relevant moderator and thus should be included in studies on text generation.

#### Summary: heterogeneous findings and moderating factors

In sum, the available meta-analyses reported heterogenous findings for various moderators that might affect the occurrence and magnitude of the text generation effect. Two of them included only studies with less complex learning material (Bertsch et al., 2007; McCurdy et al., 2020), and the meta-analysis by Schindler and Richter (2023) was based on only 20 studies and tested these moderators across genres and generation tasks but not separately for studies in which learners unscrambled expository texts. Thus, a systematic investigation of moderating factors affecting the text generation effect for learning with expository texts is still desirable.

### Aims of the present study

The aim of the present study was to test the replicability of the text generation effect for unscrambling sentences in expository texts while systematically varying contextual factors that—based on extant literature—can be assumed to affect the occurrence and magnitude of the text generation effect. To this aim, seven experiments were conducted in which participants either read (control condition) or unscrambled sentences (generation condition) in expository texts. The experiments varied systematically on four potential moderators. In five experiments, participants were informed about the upcoming learning test (intentional learning), whereas they were naïve to the test in two experiments (incidental learning). In three experiments, learning time was constrained to 2 min and in two experiments to 3 min to ensure that learners in both groups spent the same amount of time with the learning material to avoid possible generation effects being attributed to time-on task. However, to compare the results to those of extant studies, which usually had no time constraints (e.g., Einstein et al., 1984, 1990; McDaniel et al., 1986, 2002), learning time was unrestricted in two of the seven experiments. In these studies, individual learning time was included as a covariate in the analyses. This method should clarify whether a pre-set learning time leads to different processing methods compared to no time.

Given that desirable difficulties should especially foster long-term learning (Bjork & Bjork, 2011), the learning test took place 30 min after learning in one of the experiments and after one week in another. The learning test took place immediately after learning in all remaining experiments. Finally, learning condition was varied between-subjects in six experiments and within-subjects in one experiment.

Another aim was to increase ecological validity of the study by using authentic psychology textbook learning material, which was important for participant’s exam preparation in four of the seven experiments. Moreover, participants worked on multiple texts instead of one to ensure generalizability of the findings. Two learning tests were designed in accordance with actual exam question formats (prompted recall and multiple-choice questions). All data were analyzed using generalized mixed models to account for the nested data structure of the experimental design, which had a crossed random effects structure with participants nested within items and items nested within participants.

According to the findings of the meta-analysis by Schindler and Richter (2023), a positive generation effect is to be expected in all seven experiments. However, no clear expectations could be derived for differences in magnitude between intentional and incidental learning, between different types of retention interval, learning time constraint, study design, and learning tests based on the extant literature.

## Methods

All experiment and sample characteristics are reported in Table 1. The following sections describe the samples and general procedure across all seven experiments and the procedural differences between experiments.

**Table 1:** Overview of experiments and sample characteristics

	Inten-tiona-lity	Time cons-traint	Retention interval	Design	Learning assessment test	*N*	Gender (f/m)	Age *M*(*SD*)	Sample	Remunera-tion or credit
Experiment 1	int	2 min	Immediate	between-subjects	Prompted recall	40	25/15	29.07 (10.89)	Teaching students & mixed sample	Course credit or 10,-€
Experiment 2	int	none	Immediate	between-subjects	Prompted recall MC-questions (A)	61	40/21	28.13 (10.80)	Mixed sample	12,-€
Experiment 3	inc	2 min	Immediate	between-subjects	Prompted recall MC-questions (B)	94	74/20	20.10 (2.57)	Teaching students	Course credit + 3,-€
Experiment 4	int	2 min	30 min	between-subjects	Prompted recall MC-questions (A)	63	49/14	22.44 (6.80)	Teaching students & mixed sample	Course credit + 5,-€ or 15,-€
Experiment 5	int	3 min	Immediate	within-subjects	Prompted recall MC-questions (C)	79	57/22	24.70 (6.16)	Psychology students & mixed sample	15,-€
Experiment 6	inc	none	Immediate	between-subjects	Prompted recall MC-questions (C)	152	89/35 mis: 28	25.80 (7.72)	Mixed sample	15,-€
Experiment 7	int	3 min	1 week	between-subjects	Prompted recall MC-questions (C)	142	111/31	22.46 (5.56)	Teaching students & mixed sample	Course credit or 15,-€

Intentionality: int = intentional learning, inc = incidental learning (participants were naïve to the learning test); time constraint: learning time constraint per paragraph; learning assessment test: three different versions of the multiple-choice questionnaire (A, B, and C) were administered; gender: sociodemographic information is missing for 28 participants in Experiment 6 because of a technical error; all experiments were conducted at the University of Würzburg, Germany; Experiment 6 was also partially conducted at the University of Kassel, Germany. Please note that mixed samples also included non-students (Experiment 1: *n* = 11; Experiment 2: *n* = 13; Experiment 3: *n* = 0; Experiment 4: *n* = 3; Experiment 5: *n* = 12; Experiment 6: *n* = 15; Experiment 7: *n* = 12)

### Samples

All participants were recruited between 2017 and 2022 at the University of Würzburg, Germany except for Experiment 6 for which participants were partially recruited at the University of Kassel, Germany. Participants were excluded from the analyses in case of technical difficulties (Experiment 2: *n* = 2; Experiment 3: *n* = 1; Experiment 5: *n* = 2; Experiment 6: *n* = 1), if they did not adhere to the instructions (Experiment 4: *n* = 2; Experiment 5: *n* = 1; Experiment 7: *n* = 2) or if they did not participate in the learning test (Experiment 7: *n* = 15). Final sample sizes ranged from 40 (Experiment 1) to 152 participants (Experiment 6), and all participants gave their written consent.

Experiment 3 was conducted with teaching students only, and in Experiments 1, 4, and 7 teaching students were just part of the sample. The learning material was relevant for their end-of-the-semester exam, and they received course credit for their participation. Teaching students in Experiment 3 received course credit plus a small monetary remuneration because they had reached their course credit limit. All teaching students were recruited in the University courses of the first author. All other participants (Psychology students or mixed samples which also included non-students) were recruited via the participant pool management system Sona (Sona Systems, n.d.) at both universities. The settings of Sona were set to ensure that no participant took part in more than one of the experiments. They received monetary remuneration depending on the experiment’s duration which ranged from about one hour (Experiment 1) to about 1.5 h (Experiments 4 to 7).

Experiments 1 to 5 were available to participants for the lecture period of one semester each with Experiments 1 and 2 and Experiments 3 and 4 being conducted in parallel. Experiment 6 was conducted at two universities in two subsequent semesters, and Experiment 7 started just before the pandemic and the first lockdown and was continued across the time span of three semesters when testing in the laboratory was possible again. In all Experiments we aimed at collecting data from as many participants as possible during one semester (Experiments 1 to 5) or during the time funding was available (Experiments 6 and 7), which resulted in varying sample sizes.

The mean age ranged from 20.10 years (Experiment 2) to 29.07 years (Experiment 1), with female participants being overrepresented in all seven experiments.

*Power analyses*. According to extant literature, large text generation effect sizes should be expected. McDaniel et al. (1986) report *F* statistics that correspond to large effects sizes (Experiment 1: *f* = 0.61; Experiment 2: *f* = 1.179), similar to other relevant studies, for example Einstein et al., (1990, Experiment 1: *f* = 0.5405) and McDaniel et al., (2002, Experiment 1B: *f* = 0.4465 for high ability readers and *f* = 0.427 for low ability readers, Experiment 2B: *f* = 0.38 for high ability readers and *f* = 0.515 for low ability readers). Sample sizes required to detect comparable effects in the present study were estimated using G*Power (a priori analyses for Experiments 3, 5, 6, and 7; Experiments 3 and 4 were conducted in parallel with Experiment 4 starting a few weeks earlier). For achieving a power (1 − β) of 0.95 (at α = 0.05), required sample sizes for a two-group comparison in a between-subjects design ranged from 12 participants based on the effect sizes reported by McDaniel et al., (1986, Experiment 2) to 92 participants based on McDaniel et al., (2002, Experiment 2B). For achieving a power of 0.80, they ranged from 10 participants based on McDaniel et al., (1986, Experiment 2) to 32 participants based on McDaniel et al., (2002, Experiment 2B). All seven experiments in the present study should thus have sufficient power based on the reported effect sizes of McDaniel et al. (1986) at a power of 95%, and at a power of 80% when estimates were based on Einstein et al., (1990, Experiment 1). The power of Experiment 5 should be even higher than the power of the other experiments because learning condition was varied within-subjects in this experiment (Cohen, 2013).

### Material and procedure

All seven experiments were conducted in a laboratory on desktop computers.

#### Text material

In all seven experiments, participants either unscrambled sentences (generation condition) or read (reading control condition) 12 short texts containing of six sentences each. All texts were taken (and slightly modified) from a psychology textbook chapter on Bandura’s social-cognitive learning theory (Mazur, 2006) because the texts were all relevant for the exam of the teaching students, and the texts also ensured comparable text difficulty. The mean Flesch reading ease according to Amstad’s (1978) formula for German texts was 38.2 which corresponds to the difficulty level of a university text (*SD* = 7.7, *Min* = 24.9, *Max* = 47,3; only two of the twelve texts had the highest difficulty level of academic texts). All texts were modified in a way that each text dealt with a separate and coherent sub-topic (e.g., the person Albert Bandura, motivational aspects of social learning, inhibition effect) and that each text contained six sentences. Text length varied between 97 and 130 words (*M* = 109.33, *SD* = 10.95). Participants were presented with one text at a time and asked to read the intact texts in the reading control condition or to reorder the scrambled sentences of each paragraph in the generation condition. Texts in the generation condition were scrambled in a way that no sentence appeared in its original position and two adjacent sentences were never presented next to each other.

#### Variation of moderators

Learning was either intentional (Experiments 1, 2, 4, 5, and 7) or incidental with participants being naïve to the upcoming learning test (Experiments 3 and 6). Learning time was restricted to 2 min per text in Experiments 1, 3, and 4 and restricted to 3 min in Experiments 5 and 7, and no restriction was imposed in Experiments 2 and 6. Individual learning time was recorded in experiments with incidental learning to be statistically controlled in the analyses. The retention interval between learning and learning test varied between immediate testing (Experiments 1, 2, 3, 5, and 6), 30 min (Experiment 2), and 1 week (Experiment 7). Learning condition was varied between subjects (each participant either read or generated all 12 texts, Experiments 1 to 4 and 6 to 7) or within subjects (each participant read six texts and generated six texts, with the order generate–read and read-generate being varied between subjects, Experiment 5). Participants were randomly assigned to experimental groups in all between-subjects experiments based on their grade on their final school exam (i.e., German Abitur for most participants). Participants were assigned in a way that grade means, range, and standard deviation were comparable across groups as a proxy for comparable reading abilities.

#### Distraction task

A short distraction task, which comprised the Need for Cognition Scale (33 items, Bless et al., 1994), was then administered, followed by two learning tests. Although the NFC scale was originally meant to be a distractor task in the present study it was treated as potential covariate later on.

#### Prompted recall

Learning was assessed via a prompted recall test in all seven experiments in which the participants were asked to recall as much information from each paragraph as possible. Twelve questions (one per text) prompted each subtopic and required the participants to write down everything they could remember on a specific sub-topic (e.g., *Please write down everything you know about Albert Bandura*) and sometimes additionally asked them to provide classroom examples which required real understanding of the texts (e.g., *What is meant by motivational processes? Illustrate this with an example of your choice from everyday classroom life*).

#### MC-questionnaire

Participants also worked through 20 multiple-choice (MC) questions containing text-based questions (asking for information which was explicitly stated in the texts) and inference questions (asking for knowledge that had to be inferred based on participants’ text knowledge). Each MC question had four response options of which either one, two, three, or all four could be correct. No response option ruled out the other. The incorrect response options were designed either to contradict information explicitly stated in the text or to be ruled out as incorrect based on inferred information. There were three versions of the MC-test (A: Experiments 2 and 4, B: Experiment 3, C: Experiments 5 to 7) because the questions were optimized over the period of investigation. The revisions of the MC test primarily concerned the number of text-based and inference questions. Whereas Version A included mostly text-based questions, the first revision (Version B) contained 8 inference questions out of 20, and the final Version C contained an equal number of text-based (10) and inference questions (10). In addition, the MC questions in Version C were adjusted so that each individual response option could be unambiguously assigned to a specific text passage. This was necessary to determine for each learner which response option referred to a generated versus a read text passage in the analysis of the within-subjects Experiment 5. The MC questions for all three versions are provided in the Supplemental Material (Tables 3, 4 and 5).

#### Questionnaire

At the end of each experiment, participants filled in a questionnaire assessing several control variables such as prior knowledge (*yes*/*no*), native speaker status (*yes*/*no*), learning motivation (7-point Likert-scale: 1 = *not motivated at all* to 7 = *very motivated*), generation and/or reading motivation (1 = *not motivated at all* to 7 = *very motivated*), test performance motivation (1 = *not motivated at all* to 7 = *very motivated*), topic interest (1 = *not motivated at all* to 7 = *very motivated*), learning strategy use (*yes*/*no*), generation strategy use (*yes*/*no*; only those who generated texts), and test expectancy in experiments with incidental learning (*test expected*/*not expected*). Considering that the learning test took place after a 1-week delay in Experiment 7, we also asked participants whether they voluntarily or involuntarily prepared for the learning test by getting more information on the topic (*yes*/*no*), which they had been asked explicitly not to do. We also asked them whether they had purposefully used specific memory strategies (*yes*/*no*). When asked for learning strategies, generation strategies, and memory strategies, participants also could indicate in an open text field which individual strategies they had used (in four of six experiments). In case experimental groups differed in one or more of these control variables, they were included as covariates in the analyses.

Finally, all participants were asked to rate overall text comprehensibility (1 = *not comprehensible at all* to 7 = *very comprehensible*). This question served as a manipulation check. Given that text generation is conceptualized as a desirable difficulty, generated texts are assumed to be perceived as less comprehensible than read texts. Note that comprehensibility was assessed for each individual text as a cover task in incidental learning Experiments 3 and 6. In Experiment 5 (learning condition varied within-subjects), participants reported overall text comprehensibility separately for generated and read texts. All questions are listed in the Supplemental Material (Supplemental Material Table 1).

None of the experiments were pre-registered.

## Results

We first describe the coding procedures for the participants’ open answers in the prompted recall test and for their MC-test responses. Subsequently, we report results on generation accuracy and results of tests for group differences. Finally, generalized linear mixed model (GLMM) results are reported for answers in the prompted recall and MC-test. All statistical significance tests were based on a Type-I error rate of 0.05 (two-tailed).

### Coding

All answers to the 12 prompted recall questions were coded by three independent raters. To this aim, the texts were broken into idea units (propositions). One point was awarded for each correctly recalled idea unit. No point was awarded for incorrect or no recall. Partial points were not awarded. Also, participants received an additional point for each correctly provided classroom example. They could reach a maximum of 51 points for the prompted recall test. Inter-rater reliability was estimated for the three raters in each experiment. Inter-rater reliability was high for Experiments 1 to 6 and moderate for Experiment 7 (Table 2). To increase measurement accuracy, all three ratings were collapsed into a majority rating. That is, for each person and idea unit, the rating that was provided by at least two of the three raters was used. For two experiments single ratings were missing though. In Experiment 1, 32 of 2,040 data points (1.6%) were missing for one rater, and in Experiment 7 one rater failed to code the answers from 40 of the 142 participants (28%). In most of those cases, the remaining ratings were congruent and thus constituted the majority rating. In case the two raters disagreed, the coding of the rater who showed an overall higher inter-rater reliability with the rater whose ratings were missing was used for the specific idea unit. Descriptive statistics for mean proportions of correctly recalled idea units in the prompted recall test (majority rating) are presented in Table 2.

**Table 2:** Mean proportions of correctly recalled idea units across learners per paragraph and across paragraphs

	*N*	P1 *M*(*SD*)	P2 *M*(*SD*)	P3 *M*(*SD*)	P4 *M*(*SD*)	P5 *M*(*SD*)	P6 *M*(*SD*)	P7 *M*(*SD*)	P8 *M*(*SD*)	P9 *M*(*SD*)	P10 *M*(*SD*)	P11 *M*(*SD*)	P12 *M*(*SD*)	Across Para-graphs *M*(*SD*)
Experiment 1														
Total	40	0.38(.25)	0.51(0.22)	0.32(0.22)	0.30(0.25)	0.33(0.31)	0.29(0.40)	0.23(0.23)	0.27(0.22)	0.56(0.29)	0.53(0.35)	0.18(0.37)	0.18(0.20)	0.34(0.31)
Generation	18	0.29(0.22)	0.56(0.20)	0.27(0.26)	0.23(0.27)	0.27(0.33)	0.31(0.42)	0.24(0.28)	0.26(0.22)	0.53(0.31)	0.48(0.38)	0.06(0.24)	0.19(0.21)	0.31(0.31)
Reading	22	0.45(0.25)	0.48(0.23)	0.36(0.18)	0.35(0.23)	0.38(0.29)	0.28(0.40)	0.23(0.19)	0.27(0.22)	0.58(0.27)	0.58(0.33)	0.27(0.43)	0.17(0.20)	0.37(0.30)
Interrater-reliability		*K* = 0.632 (95% CI, 0.607 to 0.658), *p* <.001												
Experiment 2														
Total	61	0.35(0.25)	0.47(0.22)	0.40(0.25)	0.36(0.30)	0.25(0.27)	0.33(0.43)	0.23(0.22)	0.34(0.31)	0.50(0.28)	0.56(0.33)	0.30(0.43)	0.19(0.20)	0.36(0.32)
Generation	30	0.28(0.21)	0.48(0.25)	0.43(0.25)	0.36(0.30)	0.25(0.25)	0.45(0.45)	0.25(0.23)	0.36(0.31)	0.50(0.26)	0.51(0.35)	0.30(0.45)	0.22(0.20)	0.37(0.31)
Reading	31	0.42(0.26)	0.46(0.21)	0.37(0.25)	0.37(0.31)	0.24(0.29)	0.21(0.38)	0.20(0.22)	0.32(0.32)	0.51(0.30)	0.61(0.31)	0.31(0.42)	0.16(0.19)	0.35(0.32)
Interrater-reliability		*K* = 0.708 (95% CI, 0.688 to 0.729), *p* <0.001												
Experiment 3														
Whole sample														
Total	94	0.29(0.24)	0.51(0.23)	0.36(0.23)	0.27(0.24)	0.24(0.27)	0.35(0.41)	0.17(0.25)	0.37(0.31)	0.51(0.28)	0.39(0.33)	0.26(0.39)	0.18(0.21)	0.32(0.31)
Generation	47	0.20(0.15)	0.46(0.24)	0.31(0.22)	0.21(0.24)	0.19(0.25)	0.29(0.36)	0.16(0.18)	0.30(0.27)	0.48(0.28)	0.29(0.27)	0.17(0.32)	0.18(0.19)	0.27(0.27)
Reading	47	0.38(0.27)	0.55(0.22)	0.40(0.24)	0.330.24	0.30(0.27)	0.41(0.45)	0.19(0.30)	0.43(0.33)	0.54(0.28)	0.49(0.35)	0.34(0.43)	0.18(0.23)	0.38(0.33)
Test not expected														
Total	59	0.25(0.22)	0.49(0.23)	0.33(0.22)	0.23(0.24)	0.21(0.25)	0.32(0.38)	0.17(0.23)	0.33(0.30)	0.49(0.30)	0.35(0.31)	0.23(0.39)	0.18(0.20)	0.30(0.30)
Generation	37	0.19(0.14)	0.46(0.24)	0.32(0.21)	0.20(0.24)	0.19(0.25)	0.33(0.36)	0.14(0.17)	0.31(0.27)	0.49(0.29)	0.29(0.26)	0.16(0.31)	0.18(0.20)	0.27(0.27)
Reading	22	0.36(0.29)	0.53(0.22)	0.35(0.25)	0.29(0.25)	0.25(0.26)	0.30(0.41)	0.22(0.31)	0.36(0.35)	0.50(0.33)	0.45(0.36)	0.34(0.47)	0.17(0.20)	0.34(0.33)
Interrater-reliability		*K* = 0.720 (95% CI, 0.704 to 0.736), *p* <.001												
Experiment 4														
Total	63	0.39(0.20)	0.48(0.22)	0.41(0.25)	0.30(0.27)	0.30(0.28)	0.35(0.42)	0.12(0.21)	0.38(0.34)	0.50(0.30)	0.48(0.36)	0.33(45)	0.12(0.20)	0.35(0.32)
Generation	31	0.32(0.19)	0.48(0.19)	0.38(0.27)	0.26(0.28)	0.26(0.26)	0.23(33)	0.10(0.18)	0.35(0.37)	0.44(0.31)	0.35(0.36)	0.26(0.41)	0.10(0.18)	0.29(0.31)
Reading	32	0.45(0.20)	0.49(0.26)	0.43(0.23)	0.34(0.26)	0.34(0.30)	0.47(0.46)	0.13(0.24)	0.40(0.30)	0.57(0.29)	0.59(0.32)	0.41(0.48)	0.14(0.22)	0.40(0.33)
Interrater-reliability		*K* = 0.694 (95% CI, 0.674 to 0.714), *p* <.001												
Experiment 5														
Total	79	0.47(0.29)	0.50(0.23)	0.42(0.26)	0.45(0.33)	0.36(0.30)	0.39(0.45)	0.24(0.27)	0.46(0.29)	0.52(0.27)	0.70(0.35)	0.46(0.47)	0.18(0.22)	0.43(0.34)
Generation	79	0.37(0.28)	0.52(0.22)	0.37(0.27)	0.41(0.33)	0.28(0.29)	0.39(0.44)	0.16(0.23)	0.46(0.32)	0.50(0.29)	0.69(0.36)	0.46(0.50)	0.22(0.22)	0.40(0.35)
Reading	79	0.58(0.26)	0.48(0.25)	0.48(0.25)	0.50(0.33)	0.44(0.30)	0.39(0.45)	0.31(0.28)	0.47(0.26)	0.54(0.26)	0.71(0.35)	0.46(0.45)	0.14(0.21)	0.46(0.34)
Interrater-reliability		*K* = 0.663 (95% CI, 0.645 to 0.681), *p* <.001												
Experiment 6														
Total	152	0.19(0.16)	0.46(0.24)	0.29(0.25)	0.28(0.23)	0.20(0.24)	0.22(0.35)	0.16(0.22)	0.32(0.29)	0.42(0.32)	0.49(0.37)	0.15(0.32)	0.21(0.19)	0.28(0.29)
Generation	75	0.18(0.17)	0.52(0.25)	0.29(0.26)	0.25(0.23)	0.19(0.25)	0.24(0.36)	0.17(0.22)	0.37(0.29)	0.40(0.32)	0.43(0.34)	0.17(0.36)	0.24(0.20)	0.29(0.30)
Reading	77	0.20(0.15)	0.41(0.21)	0.29(0.24)	0.30(0.24)	0.20(0.24)	0.20(0.35)	0.15(0.23)	0.28(0.28)	0.43(0.33)	0.54(0.39)	0.14(0.28)	0.19(0.18)	0.28(0.29)
Interrater-reliability		*K* = 0.626 (95% CI,.614 to.639), *p* <.01												
Experiment 7														
Total	142	.22(.17)	.55(.25)	.35(.27)	.38(.28)	0.23(0.27)	0.09(0.22)	0.25(0.23)	0.23(0.27)	0.43(0.31)	0.46(0.39)	0.08(0.25)	0.24(0.21)	0.29(0.30)
Generation	71	0.21(0.16)	0.53(0.26)	0.31(0.27)	0.32(0.28)	0.16(0.23)	0.08(0.22)	0.21(0.20)	0.19(0.26)	0.43(0.28)	0.32(0.37)	0.01(0.12)	0.22(0.20)	0.25(0.28)
Reading	71	0.23(0.17)	0.58(0.23)	0.39(0.25)	0.44(0.27)	0.30(0.29)	0.09(0.23)	0.29(0.26)	0.28(0.27)	0.43(0.34)	0.61(0.35)	0.14(0.33)	0.25(0.22)	0.34(0.31)
Interrater-reliability		*K* = 0.577 (95% CI, 0.562 to 0.539), *p* <.001												

Maximum number of idea units per paragraph: T1 = 7, T2 = 4, T3 = 5, T4 = 5, T5 = 5, T6 = 4, T7 = 4, T8 = 3, T9 = 4, T10 = 5, T11 = 2, T12 = 3, Sum = 51; learning condition was varied within subjects in Experiment 5; individual ratings were missing for one of the three raters in Experiments 1 and 7, thus, interrater- reliability was estimated only for complete (i.e., with three) ratings in Experiments 1 and 7. Fleiss’ Kappa was estimated according to Fleiss (1971).Maximum number of idea units per paragraph: T1 = 7, T2 = 4, T3 = 5, T4 = 5, T5 = 5, T6 = 4, T7 = 4, T8 = 3, T9 = 4, T10 = 5, T11 = 2, T12 = 3, Sum = 51; learning condition was varied within subjects in Experiment 5; individual ratings were missing for one of the three raters in Experiments 1 and 7, thus, interrater- reliability was estimated only for complete (i.e., with three) ratings in Experiments 1 and 7. Fleiss’ Kappa was estimated according to Fleiss (1971)

For the MC questions, each MC option correctly selected and each option correctly not selected was awarded with one point. Falsely selected or unselected options were awarded with no points. Participants could reach a maximum score of four points per question and 80 points in total. Descriptive statistics for MC-test performance are presented in Table 3.

**Table 3:** MC- Scores (Proportions) per learning condition and for text-based and inference questions (Experiments 3–7)

	*N*	MC-Scores		
		Total	Text-based	Inference
Experiment 2				
*M*_total_(*SD*)	61	0.79(0.09)	–	–
*M*_gen_(*SD*)	30	0.81(0.09)	–	–
*M*_read_(*SD*)	31	0.78(0.08)	–	–
	Cronbach’s α = 0.78 (questionnaire Version A)			
Experiment 3				
Whole sample				
*M*_total_(*SD*)	94	0.75(0.09)	0.89(0.11)	0.61(0.09)
*M*_gen_(*SD*)	47	0.73(0.09)	0.87(0.11)	0.59(0.09)
*M*_read_(*SD*)	47	0.77(0.08)	0.91(0.11)	0.62(0.08)
Test not expected				
*M*_total_(*SD*)	59	0.73(0.09)	0.87(0.12)	0.58(0.09)
*M*_gen_(*SD*)	37	0.72(0.09)	0.87(0.11)	0.58(0.09)
*M*_read_(*SD*)	22	0.73(0.10)	0.88(0.13)	0.59(0.09)
	Cronbach’s α = 0.75 (questionnaire Version B)			
Experiment 4				
*M*_total_(*SD*)	63	0.81(0.07)	–	–
*M*_gen_(*SD*)	31	0.80(0.08)	–	–
*M*_read_(*SD*)	32	0.82(0.06)	–	–
	Cronbach’s α = 0.72 (questionnaire Version A)			
Experiment 5				
*M*_total_(*SD*)	79	0.81(0.11)	0.80 (0.12)	0.83(0.11)
	Cronbach’s α = 0.87 (questionnaire Version C)			
Experiment 6				
Whole sample				
*M*_total_(*SD*)	152	0.78(0.09)	0.74(0.10)	0.82(0.10)
*M*_gen_(*SD*)	75	0.78(0.08)	0.73(0.09)	0.82(0.10)
*M*_read_(*SD*)	77	0.78(0.09)	0.74(0.10)	0.81(0.10)
Test not expected				
*M*_total_(*SD*)	119	0.78(0.09)	0.73(0.10)	0.81(0.10)
*M*_gen_(*SD*)	63	0.78(0.08)	0.74(0.09)	0.83(0.09)
*M*_read_(*SD*)	56	0.77(0.10)	0.73(0.11)	0.80(0.11)
	Cronbach’s α = 0.76 (questionnaire Version C)			
Experiment 7				
*M*_total_(*SD*)	142	0.79(0.07)	0.75(0.08)	0.83(0.08)
*M*_gen_(*SD*)	71	0.79(0.06)	0.75(0.07)	0.82(0.08)
*M*_read_(*SD*)	71	0.80(0.08)	0.76(0.09)	0.84(0.08)
	Cronbach’s α = 0.65 (questionnaire Version C)			

A total of 80 points could be achieved in the multiple-choice test; there were no multiple-choice questions in Experiment 1; learning condition was varied within subjects in Experiment 5; the multiple-choice questions could only be clearly assigned to the categories text-based and inference questions in Experiments 3, 5, 6, and 7

### Generation accuracy

Generation accuracy was operationalized as the mean deviation of a sentence from its original position in a text after unscrambling. This deviation score (higher scores indicate less accurate generation) was calculated according to McDaniel et al. (1986) and Schindler et al. (2024). The mean deviation score of the scrambled texts was 2.25. In all seven experiments the unscrambled texts had significantly lower deviation scores than the scrambled texts (Experiment 1: *t*(17) = − 15.71, *p* <.001; Experiment 2: *t*(29) = − 22.78, *p* <.001; Experiment 3: *t*(46) = − 31.87, *p* <.001; Experiment 4: *t*(30) = − 25.06, *p* <.001; Experiment 5: *t*(78) = − 39.51, *p* <.001; Experiment 6: *t*(74) = − 38.95, *p* <.001; Experiment 7: *t*(70) = − 52.35, *p* <.001), indicating successful generation efforts.

In addition, a coherence score was calculated indicating how well a person identified adjacent sentences. Each correctly identified sentence pair was awarded with one point and means were calculated. Scores could range from 0 to 1 with higher scores indicating higher generation accuracy. Again, coherence was significantly higher after unscrambling in all seven experiments than for the scrambled texts, which was 0 (Experiment 1: *t*(17) = 16.23, *p* <.001; Experiment 2: *t*(29) = 21.04, *p* <.001; Experiment 3: *t*(46) = 31.68, *p* <.001; Experiment 4: *t*(30) = 30.42, *p* <.001; Experiment 5: *t*(78) = 31.92, *p* <.001; Experiment 6: *t*(74) = 35.16, *p* <.001; Experiment 7: *t*(70) = 45.57, *p* <.001). Descriptive statistics for the deviation and coherence scores are presented in Table 4.

**Table 4:** Descriptive statistics for all variables except for prompted recall and MC-Scores

	Experiment 1		Experiment 2		Experiment 3		Experiment 4		Experiment 5		Experiment 6		Experiment 7	
	Gen	Read	Gen	Read	Gen	Read	Gen	Read	Gen	Read	Gen	Read	Gen	Read
Prior knowledge														
*n*_yes_	5	5	13	5	6	7	10	7	32		11	23	29	30
*n*_no_	13	17	17	26	41	40	21	25	47		64	54	42	42
Native speaker														
*n*_yes_	16	21	30	28	46	46	29	31	71		66	45	65	66
*n*_no_	2	1	0	3	1	1	2	1	8		9	4	6	5
Deviation Score *M*(*SD*)	0.91 (0.36)	–	0.73 (0.37)	–	0.79 (0.31)	–	0.76 (0.33)	–	0.65 (0.36)		0.71 (0.34)	–	0.62 (0.26)	–
Coherence Score *M*(*SD*)	0.57 (0.15)	–	0.64 (0.17)	–	0.60 (0.13)	–	0.63 (0.11)	–	0.68 (0.19)		.64 (0.16)	–	0.68 (0.13)	–
Learning Motivation *M*(*SD*)	3.78 (1.48)	4.59 (1.65)	4.03 (1.54)	4.19 (1.60)	–	–	3.94 (1.39)	5.34 (0.90)	–		–	–	–	–
Generation/Reading Motivation *M*(*SD*	5.89 (1.08)	–	5.73 (1.46)	–	5.51 (1.40)	4.57 (1.46)	5.55 (1.51)	–	5.78 (1.46)	5.00 (1.58)	5.75 (1.04)	5.12 (1.25)	5.87 (1.19)	5.53 (1.25)
Test Performance Motivation *M*(*SD*)	3.94 (1.80)	4.09 (1.74)	4.33 (1.67)	4.26 (1.63)	4.02 (1.55)	4.49 (1.49)	3.74 (1.86)	4.66 (1.54)	4.92 (1.62)		4.39 (1.65)	4.87 (1.49)	4.73 (1.37)	5.62 (1.07)
Topic Interest *M*(*SD*)	4.50 (1.29)	5.05 (1.25)	4.80 (1.77)	4.90 (1.51)	5.13 (1.28)	5.13 (1.39)	5.32 (1.47)	5.91 (0.86)	4.86 (1.33)	4.75 (1.38)	4.85 (1.43)	5.32 (1.32)	5.49 (1.12)	5.77 (1.10)
NFC *M*(*SD*)	4.64 (.44)	4.56 (.84)	4.69 (0.85)	4.78 (0.72)	4.79 (0.57)	4.75 (0.70)	4.89 (0.82)	4.89 (0.59)	4.72 (0.64)		4.93 (0.75)	4.91 (0.67)	4.68 (0.69)	3.61 (9.73)
Learning Strategy Use														
*n*_yes_	2	6	2	7	4	13	2	15	21	33	2	11	55	
*n*_no_	16	16	28	24	43	34	29	17	58	46	72	66	15	
Generation Strategy Use														
*n*_yes_	–	–	–	–	37	–	–	–	46	–	58	–	60	–
*n*_no_	–	–	–	–	10	–	–	–	33	–	17	–	11	–
Learning time in sec	–	–	1270.43 (445.38)	568.23 (225.66)	–	–	–	–	–		1446.16 (450.39)	578.82 (159.42)	–	–
German grade in final school exam *M(SD*)	9.33 (2.14)	10.59 (2.20)	9.50 (2.30)	9.45 (1.84)	9.45 (2.63)	9.13 (2.82)	9.76 (2.81)	9.45 (3.24)	–		10.24 (2.22)	10.29 (1.99)	10.17 (2.80)	10.14 (2.58)
Test expected														
*n*_yes_	–	–	–	–	10	25	–	–	–		12	21	–	–
*n*_no_	–	–	–	–	37	22	–	–	–		63	56	–	–
Preparation														
*n*_yes_	–	–	–	–	–	–	–	–	–		–	–	3	3
*n*_no_	–	–	–	–	–	–	–	–	–		–	–	68	68
Memory Strategy Use														
*n*_yes_	–	–	–	–	–	–	–	–	–		–	–	59	56
*n*_no_	–	–	–	–	–	–	–	–	–		–	–	12	15
Text Comprehensibility	4.56 (1.15)	5.77 (1.23)	5.73 (1.23)	5.26 (1.32)	5.70 (0.74)	6.31 (0.66)	5.19 (1.38)	6.09 (0.78)	5.67 (1.33)	5.89 (1.27)	6.17 (0.98)	6.88 (0.65)	6.10 (1.02)	6.59 (0.75)

Comprehensibility ratings were assessed individually for each paragraph (instead of across paragraphs) as cover task in the incidental learning Experiments 3 and 6 (displayed in the table: mean comprehensibility ratings across paragraphs separately for generated and read paragraphs); learning time was assessed only in experiments without learning time constraint; German grades (not accessed in Experiment 5) could range from 0 to 15 points with 15 points being an excellent grade; deviation score: mean deviation of the final sentence’s position (after unscrambling) from its original position (low scores mean more successful unscrambling); coherence score: indication of how often a learner identified two adjacent sentences (scores could range from 0 to 1 with scores close to 1 being better); learning condition was varied within subjects in Experiment 5; due to a technical error, demographics are missing for 28 participants (reading condition) in Experiment 6

### Tests for group differences

Descriptive statistics for potential control variables and text comprehensibility as manipulation check are presented in Table 4. Note that questionnaires changed slightly across experiments and that not all variables were assessed in all seven experiments. The results of the tests for group differences are reported in Table 5. All control variables that differed significantly between experimental groups were statistically controlled in the GLMMs, which are reported in the next sections. Open answers to strategy questions were not provided consistently and differed very much in quality. In the context of the present study, these answers are therefore used only to inform the discussion of the focal results. They are provided though in the data files on OSF [https://osf.io/w9gks/?view_only=3c911a39e55049dbab42756abb310896].

**Table 5:** Tests for group differences in control variables, text comprehensibility (manipulation check), and interrater reliability

	Experiment 1	Experiment 2	Experiment 3	Experiment 4	Experiment 5	Experiment 6	Experiment 7
Prior knowledge	χ^2^(1, *N* = 40) = 0.14, *p* =.714	χ^2^(1, *N* = 61) = 5.42, *p* =.020	χ^2^(1, *N* = 94) = 0.89, *p* = .765	χ^2^(1, *N* = 63) = 0.86, *p* =.353	–	χ^2^(1, *N* = 152) = 5.06, *p* =.025	χ^2^(1, *N* = 142) = 0.03, *p* = .865
Native speaker	χ^2^(1, *N* = 37) =.615, *p* =.579	χ^2^(1, *N* = 61) = 3.05, *p* =.081	χ^2^(1, *N* = 94) = 0.00, *p* = 1.000	χ^2^(1, *N* = 63) = 0.384, *p* =.535	–	χ^2^(1, *N* = 124) = 0.47, *p* =.495	χ^2^(1, *N* = 142) = 0.10, *p* = .754
Learning Motivation	*t*(38) =− 1.62, *p* =.113	*t*(59) = − 0.40, *p* =.692	–	*t*(51.24) = − 4.76, *p* <.001	–	–	–
Generation/Reading Motivation	–	–	*t*(92) = 3.18, *p* =.002	–	*t*(78) = 3.84, *p* <.001	*t*(150) = 3.38, *p* <.001	*t*(139) = 1.68, *p* =.096
Test Performance Motivation	*t*(38) =− 0.26, *p* =.796	*t*(59) = 0.18, *p* =.692	*t*(92) =− 1.49, *p* =.139	*t*(61) =− 2.13, *p* =.037	–	*t*(150) =− 1.90, *p* =.060	*t*(132.33) =− 4.29, *p* <.001
Topic Interest	*t*(38) =− 1.35, *p* =.185	*t*(59) =− 0.25, *p* =.807	*t*(92) = 0.00, *p* = 1.000	*t*(47.96) =− 1.92, *p* =.061	*t*(78) = 0.82, *p* =.417	*t*(150) =− 2.11, *p* =.036	*t*(140) =− 1.51, *p* =.132
NFC	*t*(38) = 0.41, *p* =.684	*t*(59) =− 0.47, *p* =.643	*t*(92) = 0.30, *p* =.762	*t*(61) =− 0.03, *p* =.976	–	*t*(150) = 0.17, *p* =.864	*t*(140) = 0.92, *p* =.360
Learning Strategy Use	χ^2^(1, *N* = 40) = 1.616, *p* =.204	χ^2^(1, *N* = 61) = 3.07, *p* =.080	χ^2^(1, *N* = 94) = 5.82, *p* =.016	χ^2^(1, *N* = 63) = 13.06, *p* <.001	χ^2^(1, *N* = 79) = 4.05, *p* =.044	χ^2^(1, *N* = 152) = 4.81, *p* =.028	–
Learning time in sec	–	*t*(42.65) = 7.73, *p* <.001	–	–	–	*t*(91.83) = 15.74, *p* <.001	–
German grade in final school exam	*t*(38) =− 1.82, *p* =.076	*t*(59) = 0.09, *p* =.928	*t*(92) = 0.57, *p* =.286	*t*(58) = 0.39, *p* =.698	–	*t*(149) =− 0.12,*p* =.901	*t*(83) = 0.05, *p* =.963
Preparation	–	–	–	–	–	–	χ^2^(1, *N* = 142) = 0.00, *p* = 1.000
Memory Strategy Use	–	–	–	–	–	–	χ^2^(1, *N* = 142) = 0.41, *p* =.521
Text Comprehensibility	*t*(38) =− 3.20, *p* =.003	*t*(59) = 1.46, *p* =.151	*t*(92) =− 4.27, *p* <.001	*t*(47.05) =− 3.18, *p* =.003	*t*(78) =− 1.29, *p* =.201	*t*(127.42) = −5.24, *p* <.001	*t*(140) =− 3.29,*p* <.001

Displayed are the results of tests for group differences (generate vs. read). Only few non-native speakers took part in most of the experiments, thus χ2 estimates might not be reliable; paired-samples t-tests were used in Experiment 5 because of the within-subjects design; note that deviations in the degrees of freedom from sample size in the *t*-tests are possible due to significant Levene’s Tests for Equality of Variances or due to single missing values

### GLMMs for prompted recall

GLMMs with a logit link function for prompted recall (Dixon, 2008) were estimated to examine the replicability of the text generation effect. The models for all seven experiments were estimated and tested with the software packages lme4 (Bates et al., 2015) and lmerTest (Kuznetsova et al., 2017) for R. All models included learning condition (generation vs. reading) as contrast-coded predictor variable (− 1 = read, 1 = generate). Learning time was included as a grand-mean-centered predictor variable in Experiments 2 and 6 (unlimited learning time) to ensure that potential text generation effects cannot be attributed to time-on-task. Moreover, text expectancy was included as a dummy-coded predictor variable in the models for Experiments 3 and 6 (incidental learning), with learners who reported not having expected the learning test serving as the reference group. Moreover, we included the interaction term of test expectancy and learning condition so that the main effect for learning condition was estimated for learners who were naïve to the learning test. Deviation and coherence scores as indicators of generation accuracy were included as a grand-mean centered predictor variables in Experiment 5, the only experiment based on a within-subjects design. Moreover, we included the interaction terms of both variables with learning condition to explore the extent that the text generation effect depends on generation accuracy.

In addition, control variables were included when they were found to differ significantly between groups. Consequently, prior knowledge was included as contrast-coded predictor variable (− 1 = no, 1 = yes) in the models for Experiments 2 and 6. Generation or reading motivation was included as a grand-mean centered variable in Experiments 3, 5, and 6. Learning strategy use was included as contrast-coded predictor variable (− 1 = no, 1 = yes) in the models for Experiments 3, 4, and 6. Learning motivation was grand-mean centered and included in the model for Experiment 4. Test performance motivation was also grand-mean centered and included in the models for Experiments 4 and 7. Finally, topic interest was included as a grand-mean centered predictor variable in the model for Experiment 6. Deviation and coherence scores as indicators of generation accuracy could be included only in Experiment 5 in which learning condition was varied within-subjects. Intercepts for participants and idea units were allowed to vary randomly. A random slope (varying between subjects) for learning condition was also included in the model in Experiment 5 (within-subjects design). Random slopes (varying between idea units) for learning condition were included in all seven experiments.

The results for all seven models are displayed in Table 6. Participants recalled more idea units correctly in the reading condition compared to the generation condition in four of the seven experiments (Experiment 3: β = − 0.37,* z* = − 2.73,* p* =.006, OR = 0.69, 95%-CI [0.53, 0.90]; Experiment 4: β = − 0.23,* z* = − 1.98,* p* =.048, OR = 0.80, 95%-CI [0.64, 1.00]; Experiment 5: β = − 0.22,* z* = − 3.43,* p* <.001, OR = 0.80, 95%-CI [0.70, 0.91]; Experiment 7: β = − 0.27,* z* = − 3.41,* p* <.001, OR = 0.76, 95%-CI [0.65, 0.89]). In the remaining three experiments (Experiments 1, 2, and 6), the effects of learning condition were not significant. Thus, we found no positive text generation effect on prompted recall in any of the seven experiments.

**Table 6:** Fixed effects and variance components in the GLMM for prompted recall accuracy

Parameter	Experiment 1	Experiment 2	Experiment 3	Experiment 4	Experiment 5	Experiment 6	Experiment 7
	β (*SE*)	β (*SE*)	β (*SE*)	β (*SE*)	β (*SE*)	β (*SE*)	β (*SE*)
Fixed Effects							
Intercept	− 0.94 (0.25)***	− 0.77 (0.24)**	− 1.17 (0.27)***	− 0.87 (0.20)***	− 0.40 (0.22)*	− 0.94 (0.27)***	− 1.26 (0.22)***
Learning condition^a^	− 0.22 (0.16)	− 0.24 (0.14)	− 0.37 (0.14)**	− 0.23 (0.11)*	− 0.22 (0.07)***	0.09 (0.12)	− 0.27 (0.08)***
Learning time^b^	–	0.004 (0.002)*	–	–	–	− 0.04 (0.11)	–
Prior knowledge^a^	–	0.39 (0.14)**	–	–	–	0.22 (0.08)**	–
Test expected^c^	–	–	0.19 (0.23)	–	–	− 0.24 (0.19)	–
Generation/reading motivation^b^	–	–	0.20 (0.10)	–	0.12 (0.08)	0.14 (0.08)	–
Learning strategy use^a^	–	–	0.06 (0.15)	0.03 (0.12)	0.19 (0.08)	0.37 (0.14)**	–
Learning condition X test expected	–	–	− 0.17 (0.22)	–	–	− 0.08 (0.18)	–
Learning motivation^b^	–	–	–	0.02 (0.12)	–	–	–
Test performance motivation^b^	–	–	–	0.27 (0.11)*	–	–	0.08 (0.07)
Deviation Score^b^	–	–	–	–	− 0.06 (0.18)	–	–
Coherence Score^b^	–	–	–	–	0.62 (0.18)***	–	–
Learning condition X deviation score	–	–	–	–	− 0.04 (0.09)	–	–
Learning condition X coherence Score	–	–	–	–	0.03 (0.09)	–	–
Topic interest^b^	–	–	–	–	–	0.12 (0.08)	–
Variance Components							
Subjects	0.80 (0.90)	0.72 (0.85)	0.71 (0.85)	0.39 (0.62)	0.66 (0.81)	0.55 (0.74)	0.48 (0.69)
Learning condition (RS)	–	–	–	–	0.09 (0.29)	–	–
Idea units	1.90(1.38)	2.10(1.45)	1.98 (1.41)	1.53 (1.24)	1.83 (1.35)	2.16 (1.47)	2.17 (1.47)
Learning condition (RS)	0.02 (0.12)	0.09 (0.30)	0.07 (0.26)	0.05 (0.22)	0.04 (0.20)	0.03 (0.18)	0.06 (0.24)

^a^contrast-coded, ^b^grand mean-centered, ^c^dummy-coded; learning condition: −1 = read, 1 = generate; prior knowledge: – 1 = no, 1 = yes; test expected: 0 = no, 1 = yes; learning strategy use: − 1 = no, 1 = yes

*p <.05, **p <.01, ***p <.001 (two-tailed)

Moreover, longer learning times were associated with higher recall in Experiment 2, β = 0.004,* z* = 2.55,* p* =.011, OR = 1.00, 95%-CI [1.00, 1.01]. Participants recalled more information when they had prior knowledge compared to those without prior knowledge in Experiment 2, β = 0.39,* z* = 2.89,* p* =.005, OR = 1.48, 95%-CI [1.13, 1.93], and in Experiment 6, β = 0.22,* z* = 2.61,* p* =.009, OR = 1.25, 95%-CI [1.06, 1.47]. Participants also recalled more information when they used a learning strategy than when they did not in Experiment 5, β = 0.19, *z* = 2.28, *p *=.023, OR = 1.21, 95%-CI [1.03,1.42] and in Experiment 6, β = 0.37,* z* = 2.67,* p* =.008, OR = 1.45, 95%-CI [1.10, 1.91]. Recall increased with test performance motivation in Experiment 4, β = 0.27,* z* = 2.53,* p* =.011, OR = 1.31, 95%-CI [1.06, 1.61]. Finally, participants who generated more successfully (as indicated by a higher coherence score) recalled more idea units in Experiment 5, β = 0.62,* z* = 3.53,* p* <.001 OR = 1.86, 95%-CI [1.32, 2.63].

### GLMMs for MC-questions

The GLMMs for the MC-test results as dependent variable were basically set up in the same way as the GLMMs for the prompted recall test. Intercepts for participants and MC response options were allowed to vary randomly. Also, random slopes for learning condition were included in the model for idea units in all seven experiments.

The results for all seven models are reported in Table 7. In Experiment 5, participants answered the MC questions significantly more accurately in the reading control condition than in the generation condition, β = − 0.08,* z* = − 2.00,* p* =.045, OR = 0.93, 95%-CI [0.86, 1.00]. In Experiment 6, participants in the generation condition answered the MC questions more accurately than participants in the reading condition, β = 0.17,* z* = 2.08,* p* =.037, OR = 1.18, 95%-CI [1.01, 1.38], which indicates a positive text generation effect for Experiment 6. Moreover, in Experiment 2, longer learning time was associated with better performance in the MC questions, β = 0.24,* z* = 2.08,* p* =.038, OR = 1.27, 95%-CI [1.01, 1.60]. Participants with prior knowledge recalled more information than those without prior knowledge in Experiment 2, β = 0.25,* z* = 2.67,* p* =.008, OR = 1.28, 95%-CI [1.07, 1.53]. Those who had expected the learning test recalled more information than those who were naïve to the learning test in Experiment 3, β = 0.29,* z* = 2.17,* p* =.030, OR = 1.34, 95%-CI [1.03, 1.74]. Participants in Experiment 6 also recalled more information when they reported to have used a learning strategy than when they did not, β = 0.26,* z* = 2.71,* p* =.007, OR = 1.29, 95%-CI [1.07, 1.56]. In the same experiment, participants with higher interest in the texts’ topic recalled more information than those with lower topic interest, β = 0.11,* z* = 2.17,* p* =.030, OR = 1.12, 95%-CI [1.01, 1.24].

**Table 7:** Fixed effects and variance components in the GLMM for the multiple-choice test

Parameter	Experiment 2	Experiment 3	Experiment 4	Experiment 5	Experiment 6	Experiment 7
	β (*SE*)	β (*SE*)	β (*SE*)	β (*SE*)	β (*SE*)	β (*SE*)
Fixed Effects						
Intercept	1.90 (0.16)***	1.29 (0.17)***	0.19 (0.27)	0.14 (0.27)	1.95 (0.18)***	1.81 (0.16)***
Learning condition^a^	− 0.15(0.12)	− 0.03 (0.08)	0.01 (0.07)	− 0.08 (0.04)*	0.17 (0.08)*	− 0.08 (0.05)
Learning time^b^	0.24(0.12)*	–	–	–	− 0.11 (0.08)	–
Prior knowledge^a^	0.25(0.09)**	–	–	–	0.11 (0.06)	–
Test expected^c^	–	0.29 (0.13)*	–	–	− 0.07 (0.12)	–
Generation/reading motivation	–	0.07 (0.06)	–	− 0.002 (0.06)	0.02 (0.05)	–
Learning strategy use^a^	–	− 0.03 (0.08)	0.09 (0.08)	– 0.02 (0.06)	0.26 (0.09)**	–
Learning condition X test expected	–	− 0.14 (0.13)	–	–	− 0.12 (0.12)	–
Learning motivation^b^	–	–	0.07 (0.08)	–	–	–
Test performance motivation^b^	–	–	0.08 (0.07)	–	–	0.02 (0.05)
Deviation score^b^	–	–	–	− 0.07 (0.14)	–	–
Coherence score^b^	–	–	–	0.06 (0.14)	–	–
Learning condition X deviation score	–	–	–	− 0.07 (0.06)	–	–
Learning condition X coherence score	–	–	–	− 0.05 (0.06)	–	–
Topic interest^b^	–	–	–	–	0.11 (0.05)*	–
Variance Components						
Subjects	0.28(0.53)	0.19 (0.44)	0.11 (0.33)	0.39 (0.62)	0.21 (0.46)	0.17 (0.41)
MC-option	1.45 (1.21)	1.43 (1.20)	5.21 (2.28)	5.18 (2.28)	1.49 (1.22)	1.73 (1.32)
Learning condition (RS)	–	0.02 (0.15)	–	–	–	0.02 (0.13)

^a^contrast-coded, ^b^grand mean-centered, ^c^dummy-coded; learning condition: − 1 = read, 1 = generate; prior knowledge: − 1 = no, 1 = yes; test expected: 0 = no, 1 = yes; learning strategy use: − 1 = no, 1 = yes

^*^p <.05, **p <.01, ***p <.001 (two-tailed)

Additional analyses were conducted to account for question type (text-based vs. inference) in the models for Experiments 3, 5, 6, and 7 (see Table 2 in the Supplemental Material). Question type was included as a contrast-coded predictor variable (− 1 = text-based, 1 = inference) and as an interaction term with learning condition. The results did not differ notably from the models without question type with the only exception being that no significant learning advantage was found for the reading group in Experiment 5. Neither the main nor the interaction effect were significant.

## Discussion

The aim of the present study was to test the replicability of the text generation effect for unscrambling sentences in expository texts while accounting for various moderators that can be assumed to affect the occurrence and magnitude of the text generation effect. To this end, seven experiments were conducted with systematic experimental manipulations of test expectation (intentional vs. incidental), learning time constraint (yes vs. no), retention interval (immediate, 30 min, 1-week delay), and study design (between-subjects vs. within-subjects). Two learning assessment tests were used, which are common in educational settings (prompted recall questions and an MC test). In four of the seven experiments, the samples included teaching students for whom the learning material was relevant for their end-of-semester exam, which increased the ecological validity of the experiments. Data were analyzed using generalized linear mixed models, which account for the hierarchical data structure.

### Discussion of prompted recall findings

In contrast to our expectations, the text generation effect was replicated in none of the experiments for prompted recall. Quite the opposite, a learning disadvantage was observed for the generation condition in four of the seven experiments, suggesting that text generation was a learning difficulty but not a desirable one. This disadvantage occurred in incidental (Experiment 3) and intentional learning settings (Experiments 4, 5, and 7), for experiments with immediate testing (Experiments 3 and 5), for testing after 30 min (Experiment 4), and after one week (Experiment 7). The disadvantage was also shown for the one experiment with within-subjects design (Experiment 5) and for experiments with a between-subjects design (Experiments 3, 4, and 7). Results were also comparable for teaching students only (Experiment 3) and for mixed samples (Experiments 4, 5, and 7). These findings are consistent with studies that also failed to evoke the text generation effect for unscrambling sentences in expository texts (e.g., McDaniel et al., 2002, Experiment 1B; Thomas & McDaniel, 2007, Experiment 1). However, they contradict studies that reported improved learning when using unscrambling expository texts (e.g., Einstein et al., 1984, Experiment 1, 1990, Experiment 2; McDaniel, 1984; McDaniel & Kerwin, 1987; McDaniel et al., 1986, Experiment 2; Schindler et al., 2024; Schindler & Richter, 2023).

Neither a generation effect nor a disadvantage for generation was found in Experiments 2 and 6, which were notably the only experiments with unrestricted learning time. The different results between experiments with and without learning time constraint suggest that learning was qualitatively different when having limited as opposed to unlimited learning time. Learners in the generation group might have felt considerable time pressure and thus consequently might have relied on time-efficient superficial heuristics to sort the sentences instead of establishing meaningful relations between them. This explanation is supported by the responses of some participants that indicated if they had used strategies and which strategies they had used for sentence reordering. (e.g., *looking for sentences starting with enumeration*; *sentences containing examples are usually placed at the end of the text; relying on linguistic intuition; ordering and rereading;* all open-ended responses can be looked up in the data files on OSF). Only few participants reported having used ordering strategies that focused on the text’s content and meaningful relations between sentences such as thematic consistency, coherence relations, or information overlap. In other words, the cognitive processes stimulated by the generation task might not have been the intended processes conducive to learning, which might have also been true for Experiments 1, 2, and 6. Even if no learning disadvantage was found for the generation group, no generation effect was obtained either. The results of the present study seem even more substantial because various potential covariates were controlled.

Why was no generation effect found even for experiments with unlimited learning time? We would like to offer two explanations for the absence of the text generation effect, both of which are consistent with the contextual framework and the material appropriate processing framework (see also Schindler et al., 2024). First, the texts or the learners might have evoked sufficient relational processing already, which would have rendered sentence unscrambling redundant in Experiments 1, 2, and 6 and (in combination with limited learning time) maybe even hindering in Experiments 3, 4, 5, and 7. Some of the open-ended responses to the question about potentially used learning strategies suggest this (e.g., *mentally summarizing the content after reading each text passage; using memory aids*; *visualizing the text contents; memorizing key words; creating examples*). It must be said though, that learners also reported the application of superficial and usually inefficient strategies (e.g., *rehearsing the text contents* or *rereading*; see e.g. Dunlosky et al., 2013), and the majority of learners reported not having used any learning strategy at all. Those open-ended responses emphasize that future research must carefully assess how learners process the texts in the control group–especially with samples consisting mostly of university students which might process texts per default in a more elaborate and strategic way than non-academic learners.

Second, the paradigm might not have sufficiently stimulated relational processing. Participant comments about their reordering strategies point in that direction. This would suggest that the instruction to reorder sentences alone might not suffice to evoke the addressed cognitive processes conducive to learning but that more explicit instructions and maybe time for practice including feedback could be necessary to stimulate relational processing. Another implication of these considerations is that the fundamental presumption of specific genres and tasks that automatically stimulate different types of processing as proposed by McDaniel et al., (1986; McDaniel & Einstein, 1989) should be subjected to thorough testing by future research. The MC-test results of Experiment 6, however, are not congruent with the explanations just discussed because the text generation effect was found as expected. The MC-test results are discussed in the following section.

### Discussion of MC-test findings

The prompted recall results are partially corroborated by the MC-test findings. In six of the seven experiments, no text generation effect was found. We found a learning advantage for the reading control group of Experiment 5, which corroborated the prompted recall results for Experiment 5. Experiment 6 was the only experiment for which a text generation effect was found. Interestingly, this experiment was the only one in which the combination of context factors was comparable to earlier studies that have shown the text generation effect for sentence unscrambling (e.g., Einstein et al., 1990; McDaniel et al., 1986, 1994, 2002) with intentional learning, no learning time constraint, immediate testing, and a between-subjects design. A close replication study of McDaniel et al.’s (1986) Experiment 2 by Schindler et al. (2024) also found evidence for a generation effect under these specific contextual conditions. The authors reported that sentence unscrambling increased learning across genres and languages (in a multi-level regression analyses) for incidental learning, in a between-subjects design, for immediate testing, and unlimited learning time. In sum, specific combinations of contextual factors might be crucial for the text generation effect to occur.

A discrepancy worth discussing is the absence of a learning advantage for the reading group between the prompted recall and MC-test results in Experiments 3, 4, and 7. This finding suggests that processes stimulated by sentence unscrambling affect both test formats differently. A likely explanation is that relational processing basically refers to establishing coherence relations between propositions or sentences, allowing learners to establish a rich mental model of the text, which is a necessary precondition for drawing inferences from the text (Kintsch, 1994). Considering that all three MC-test versions had a high proportion of inference questions, sentence unscrambling might have had a more favorable impact on the MC-test results than on the prompted recall test results. However, entering type of question (text-based vs. inference) into the MC-test models of Experiments 3, 5, 6, and 7 resulted in no notable difference. Also, the interaction effect for type of question and learning condition was not significant, again raising the question whether the sentence unscrambling task stimulated the intended cognitive processes.

### The potential role of generation accuracy

Another explanation for the absence of the text generation effect in most of the reported analyses was discussed by Schindler et al. (2024). They surmised that successful generation (i.e., high generation accuracy) is probably a necessary precondition for the text generation effect to occur (see also McDaniel & Einstein, 2005). In their cloze replication study of McDaniel et al., (1986, Experiment 2), Schindler et al. found an indication of the expected genre-by-generation task interaction when lower letter-completion accuracy scores were excluded from the analyses. They reported, though, that an extremely high accuracy was necessary to show the expected interaction and that their results should be interpreted with caution because of the remaining small sample, non-significant simple effects, and because the interaction was no longer significant after a Bonferroni-Holm correction. They concluded that high letter-completion accuracy might be necessary for text generation to benefit learning but that an exact accuracy threshold still needs to be determined. Both accuracy measures in the present study showed neither perfect nor near-perfect generation which may be attributed to the difficulty level of the texts (mean FRE = 38.2, *SD* = 7.7). The mean deviation of the six sentences per text from their original position after unscrambling was 0.62 to 0.91 (Table 4), meaning that sentences deviated from their original position by at least half a position. The coherence score, which indicated the extent that learners identified adjacent sentences, could take values from 0 to 1 with the experiment means varying between 0.57 and 0.68. No exact threshold for sentence unscrambling accuracy can be derived from the literature, but insufficient sentence unscrambling accuracy might be (at least partially) accountable for the absence of the generation effect. Essentially, text comprehension was likely to have been impaired to the extent that the texts could not be generated correctly. However, generation accuracy was included in the models of Experiment 5 as a main effect and as an interaction effect with learning condition. By this means, the impact of generation accuracy on the occurrence and magnitude of the text generation effect was directly tested. Although the coherence score had a statistically significant impact on prompted recall (the higher the generation accuracy, the more information was recalled), the interaction with learning condition was not significant, neither for prompted recall nor for the MC test. Hence, these results contradict the assumption that low generation accuracy (alone) was responsible for the absence of the text generation effect.

### The potential role of text length

Another potential moderator could be the texts’ length. In their meta-analysis on the text generation effect, Schindler and Richter (2023) reported lowest effect sizes for texts with 0 to 300 words (*g* = 0.36), largest effect sizes for texts of 301 to 600 words (*g* = 0.63), and effect sizes in between for texts with 601 to 900 words (*g* = 0.43). No significant effect has been found for texts longer than 900 words. Given the brevity of the texts in the present study (97 to 130 words), the possibility that longer texts might have evoked a text generation effect cannot be ruled out. However, based on Schindler and Richter’s (2023) meta-analytic findings, a generation effect should have occurred nevertheless, albeit with lower effect sizes.

### The potential role of prior knowledge

Finally, a certain amount of prior knowledge could be necessary for sentence unscrambling to be a beneficial learning strategy. Sentence unscrambling makes a text per definition very incoherent. To make sense of the scrambled sentences, learners must draw inferences and close coherence gaps. Both processes are crucial for learning (Graesser, et al., 1994, 1997; Kintsch, 1994; Singer et al., 1992; van den Broek et al., 2015; Van Dijk & Kintsch, 1983) and require a certain amount of prior knowledge. Evidence comes from a larger body of studies on the *expertise-reversal effect* (Kalyuga et al., 2003) or the *reversed coherence effect* (e.g. McNamara, 2001; McNamara et al., 1996). Both effects describe versions of the phenomenon that the effectiveness of learning instructions or material depends on individual levels of prior knowledge or expertise. According to both approaches, learners with low prior knowledge or expertise need more instructional guidance (Kalyuga et al., 2003) or more explicit and coherent learning material (McNamara et al., 1996) to profit from the learning process because they lack the necessary knowledge to close coherence gaps or to draw inferences. Strongly guided learning processes and highly coherent learning material, though, can even hinder learning when learners have high prior knowledge or expertise because they have to process (and even suppress) redundant information, thereby hindering efficient learning (Kalyuga et al., 2003; McNamara et al., 1996). Prior knowledge was overall low in most of the present Experiments (see Table 4) and self-reported text comprehensibility was expectedly lower in the generation than in the reading control group in Experiments 1, 3, 4, 6, and 7. Moreover, prior knowledge was statistically controlled as a covariate in Experiments 2 and 6, but a generation effect was obtained only for the MC data in Experiment 6. Untangling the role of prior knowledge remains for future research to systematically and experimentally investigate the possible interaction of prior knowledge and learning condition.

### Limitations of the present experiments

The results of the present experiments need to be interpreted with their limitations in mind. The first limitation to discuss is that only sentence unscrambling as a text generation strategy was investigated in the present study. Other paradigms, such as word completion or letter or word unscrambling, have been suggested and used elsewhere (see e.g., Abel & Hänze, 2019; Glover et al., 1982; Goverover et al., 2008, 2013, 2014) with mixed results. Word completion (cloze) tasks, for example, could also be designed in a way that specific relational processes are stimulated that have been known to foster text comprehension such as the establishment of local and global coherence (Graesser, et al., 1997; Singer et al., 1992; Van Dijk & Kintsch, 1983) or knowledge-based inferences (see e.g., Graesser et al., 1994; van den Broek et al., 2015). Such tasks might have the potential to stimulate processes conducive for learning more purposefully and effectively than sentence unscrambling.

Another limitation are the comparatively small sample sizes of Experiments 1, 2, and 4. However, all sample sizes in the present study should have been sufficient based on the reported effect sizes of McDaniel et al. (1986), even with a power as high as 95%. Sample sizes were also sufficient based on Einstein et al. (1990) when power was set to 80%. In addition, the power of Experiment 5 was increased by its within-subjects-design compared to the between-subjects designs in the other experiments (Cohen, 2013). Still, no text generation effect was found in any of the seven experiments (except for the MC-test results of Experiment 6), regardless of the sample size of the experiments. Contrary to all expectations, some of the experiments even found a significant learning advantage in favor of the reading group. This also makes low statistical power an unlikely explanation for the absence of the text generation effect.

The present study used 12 texts instead of one to test whether the results would generalize to different texts. All texts came from the same psychology textbook to ensure their comparability in terms of length, complexity, and comprehensibility. All of them addressed different subtopics of Bandura’s social-cognitive learning theory. They were also quite short (six sentences) to ensure the experiments would not become too long, which begs the question of whether the text generation effect could have been replicated with longer texts (see the results on text length in Schindler & Richter’s meta-analysis from 2023) or with different texts. Thus, caution in terms of generalizability of the reported findings is advised. A closer look into extant research, however, reveals that most of the studies that have reported beneficial learning effects for unscrambling expository texts in the past have used the same learning material (Einstein et al., 1990; McDaniel et al., 1986, 2002; Schindler et al., 2024). It therefore cannot be ruled out that the reported generation effects are material-specific. When using text generation in educational contexts, a prerequisite should be that its effectiveness is not limited to specific texts (or texts with very specific characteristics such as a specific length) but that the strategy can be applied to various text material. It would be still interesting though–from both a theoretical and a practical point of view–to reproduce this study with different learning material to shed light on the question whether the occurrence of the generation effect in earlier studies or its absence in the present study can be attributed to specific material (characteristics) and if they are generalizable.

Control variables and MC questionnaires varied somewhat between experiments, thus limiting the comparability of the seven experiments. The results, however, were quite comparable across experiments with the most notable difference in MC-test performance occurring between Experiment 5 (learning advantage in the reading control condition) and Experiment 6 (learning advantage in the generation condition). The same MC questionnaire (Version C) was used in both experiments though. Differences in control variables were, in some cases, required by specific experiment characteristics. In other cases, adjustments were made that seemed more sensible over time and with increasing experience of the authors. The control variables, however, were not the focus of the present study, and their differential assessment across experiments had no direct impact on learning because the questionnaire was always administered after the learning phase. In the case of random group differences in potential control variables, these control variables were included in the analyses. Hence, these differences are also not a likely explanation for the absence of the text generation effect.

As in most extant studies on the text generation effect (see the meta-analysis by Schindler et al., 2024), the vast majority of participants were university students in the present study. And even in the mixed samples, it seems fair to assume that the participants who have reported not being a student have been students in the past, because participants for the subject pool were typically recruited on the university campus. The samples were therefore relatively homogeneous and consisted of participants who are accustomed to reading texts in preparation for exams and who may already routinely employ specific processing strategies for learning with texts. Future research should therefore also consider other relevant learner groups, such as primary or secondary school students, or more specific learner groups such as students with special needs.

Another limitation of the present study is that no specific learner characteristics were assessed as moderators of the text generation effect. Control variables such as need for cognition, reading ability (roughly operationalized as German exam grade in the final school exam), and prior knowledge were assessed in the present study, and groups were tested for random differences, but their interaction with the learning condition was not analyzed to prevent the models from becoming too complex. The task remains for future research to systematically and experimentally investigate these potential moderators and explicitly test their effect on the occurrence and magnitude of the text generation effect.

The results of the present study suggest that a specific combination of moderators might be necessary for the text generation effect to occur. This conclusion would be perfectly in line with the contextual framework by McDaniel and Butler (2011), which states that the success of any desirable difficulty (such as text generation) depends on a complex interaction of learning material, task or processing strategy, learner characteristics, and criterial task. More combinations of moderators than the one suggested by the present study could conceivably evoke a generation effect. These interactions need to be explored by future research, and they need to be theoretically understood.

Finally, potential factors have not been considered to date in previous research that might be crucial for the effectiveness of text generation. For example, given that most learners have never used sentence reordering as a learning strategy before, they might need more specific instructions on how to effectively generate. They might also need some time to become familiar with the task to overcome a potential usage deficit. They might further need feedback about their generation success. Generation success is more obvious when completing letters in a fragmented text, whereas for sentence unscrambling, learners never know whether the original text is just hard to understand or whether they have not yet found the correct sentence order.

### Practical implications

The generation effect was not replicated in the present study except for one of the learning measures in just one of seven experiments. Many explanations were discussed for the absence of the effect. Ultimately, however, the question remains whether a learning strategy that is so demanding and presumably only leads to a learning advantage compared to simple reading under very specific conditions (unrestricted learning time, incidental learning, and immediate testing) is suitable for practical use in environments in which many contextual factors are far less controllable than in laboratory experimental studies. Furthermore, even in studies that have found or replicated the text generation effect in the past, its effectiveness has never been compared with other common learning strategies such as summarizing, concept mapping or self-testing. Therefore, the added value of text generation for contexts such as classroom teaching or self-directed learning remains questionable.

## Conclusion

The aim of the present study was to test the replicability of the text generation effect for unscrambling sentences in expository texts while systematically varying intentionality of learning, time constraint, retention interval, and study design. Seven experiments were conducted in which participants either read or unscrambled sentences in expository texts. The results of two learning tests showed either no generation effect or even demonstrated a learning advantage in the reading control condition. Just one of the seven experiments yielded a generation effect and only for the MC-test results. This experiment resembled the study of McDaniel et al. (1986), which originally reported the genre-by-generation task interaction, and the cloze replication of that study by Schindler et al. (2024). In sum, the results indicate that a generation effect is most likely to occur when learning is intentional, when learning time is unlimited, and when testing takes place immediately after learning. Moreover, other moderators such as text length and prior knowledge might affect the occurrence of the effect. Our findings suggest that the applications of text generation in educational contexts are probably rather limited. 

## Supplementary Information

Additional file 1.

## Data Availability

The datasets generated and analyzed during the current study are available on the Open Science Framework [https://osf.io/w9gks/?view_only=3c911a39e55049dbab42756abb310896]. All texts were taken from a psychology textbook on learning and behavior by Mazur (2006) and were slightly modified for the purpose of this study. The copyright is held by the publisher. The text and learning test questions are available upon request from the corresponding author.
