# Supplementary material for: Does text generation improve learning from expository text? A conceptual replication attempt
Source: Cogn Res Princ Implic. 2025 Jun 23;10:34. doi: 10.1186/s41235-025-00645-2 (PMC12185794; doi:10.1186/s41235-025-00645-2)
Supplement: Supplementary file 1 — Additional file 1. [file 41235_2025_645_MOESM1_ESM.docx]

**Supplemental Material Table 1**

*Pre-Experimental Assessment of Prior Knowledge and Post-Experimental Questionnaire across Experiments.*

| Experiments | | | | | | | Questions | | |
| --- | --- | --- | --- | --- | --- | --- | --- | --- | --- |
| 1 | 2 | 3 | 4 | 5 | 6 | 7 | Generation group/condition | Reading group/condition | |
|  | | | | | | | **Prior knowledge** | | |
| x | x | x | x | x | x | x | *Haben Sie bereits von Banduras Lerntheorie gehört oder sind mit Teilen seiner Theorie vertraut?*  [Have you ever heard of Bandura’s learning theory or are you familiar with parts of his theory?] | | |
|  |  |  |  |  |  |  | **Text comprehensibility^a^** | | |
| x | x |  |  |  |  | x | *Wie verständlich fanden Sie die Texte?*  [How understandable did you find the texts?] | | |
|  |  |  | x |  |  |  | *Wie verständlich fanden Sie die Textabschnitte zu Banduras 'Lernen am Modell'?*  [How understandable did you find the text passages on Badura’s observational learning?] | | |
|  |  |  |  | x |  |  | *Wie verständlich fanden Sie die ersten/letzten sechs Textabschnitte zu Banduras 'Lernen am Modell'?*  [How understandable did you find the first/final six text passages on Badura’s observational learning?] | | |
|  |  |  |  |  |  |  | **Topic interest** | | |
| x | x | x |  |  | x | x | *Wie interessant fanden Sie die Texte?*  [How interesting did you find the texts?] | | |
|  |  |  | x |  |  |  | *Wie interessant fanden Sie die Textabschnitte zu Banduras 'Lernen am Modell'?*  [How interesting did you find the text passages on Badura’s observational learning?] | | |
|  |  |  |  | x |  |  | *Wie interessant fanden Sie die ersten/letzten sechs Textabschnitte?*  [How interesting did you find the first/final six text passages?] | | |
|  |  |  |  |  |  |  | **Generation/reading motivation** | | |
| x | x | x |  |  | x | x | *Wie motiviert waren Sie, die Sätze in den Texten in eine sinnvolle Reihenfolge zu bringen?*  [How motivated were you to bring the texts’ sentences into a meaningful order?] | - | |
|  |  |  | x |  |  |  | *Wie motiviert waren Sie, die Sätze in den Textabschnitten zu Banduras 'Lernen am Modell' in eine sinnvolle Reihenfolge zu bringen?*  [How motivated were you to bring the texts’ sentences on Bandura’s observational learning into a meaningful order?] | - | |
|  |  |  |  | x |  |  | *Wie motiviert waren Sie, die Sätze in den ersten/letzten sechs Textabschnitten in eine sinnvolle Reihenfolge zu bringen?*  [How motivated were you to bring the sentences of the first/final six texts into a meaningful order?] | *Wie motiviert waren Sie, die ersten/letzten sechs Textabschnitte gründlich zu lesen?*  [How motivated were you to read the first/final six text passages thoroughly?] | |
|  |  | x |  |  | x | x | - | *Wie motiviert waren Sie, die Texte gründlich zu lesen?*  [How motivated were you to read the texts into thoroughly?] | |
|  |  |  |  |  |  |  | **Learning motivation** | | |
| x | x |  |  |  |  |  | *Wie motiviert waren Sie die Textinhalte zu lernen?*  [How motivated were you to learn the texts’ content?] | | |
|  |  |  | x |  |  |  | *Wie motiviert waren Sie die Textinhalte zu Banduras 'Lernen am Modell' zu lernen?*  [How motivated were you to learn the texts’ content on Badura’s observational learning?] | | |
|  |  |  |  |  |  |  | **Test motivation** | | |
| x | x |  |  |  |  |  | *Wie motiviert waren Sie die Fragen ausführlich zu beantworten?*  [How motivated were you to answer the questions thoroughly?] | | |
|  |  |  | x |  |  |  | *Wie motiviert waren Sie die Fragen zu Banduras 'Lernen am Modell' ausführlich zu beantworten?*  [How motivated were you to answer the questions on Badura’s observational learning thoroughly?] | | |
|  |  | x |  | x | x | x | *Wie motiviert waren Sie die Fragen ausführlich bzw. gründlich zu beantworten?*  [How motivated were you to answer the questions thoroughly and diligently?] | | |
|  |  |  |  |  |  |  | **Learning strategy use** | | |
| x | x |  | x |  |  |  | *Haben Sie, um sich den Inhalt der Textabschnitte besser merken zu können, Strategien angewandt?*  [Did you use any learning strategies to better memorize the texts?] | | |
|  |  |  |  |  |  | x | *-* | | *Haben Sie zum Lernen bestimmte Strategien angewandt? Wenn ja, welche?*  [Did you use any learning strategies? If yes, which ones?]] |
| x | x |  | x |  |  |  | *Was für Strategien haben Sie zum Lernen der Textabschnitte angewandt?*  [Which learning strategies did you use to learn the texts?] | | |
|  |  | x |  |  | x |  | *Falls Sie mit einem Lerntest gerechnet haben - haben Sie bestimmte Lernstrategien angewandt, um sich auf den Test vorzubereiten? Wenn ja, welche?*  [In case, you have expected the learning test – did you use any learning strategies to prepare for the test? If yes, which ones?] | | |
|  |  |  |  | x |  |  | *Haben Sie bei den ersten/letzten sechs Textabschnitten bestimmte Lernstrategien angewandt, um sich auf den Test vorzubereiten? Wenn ja, welche?*  [Did you use any learning strategies when working on the first/final six text passages to prepare for the test? If yes, which ones?] | | |
|  |  |  |  |  |  |  | **Generation strategy use** | | |
|  |  | x |  |  | x |  | *Haben Sie beim Sortieren der Textabschnitte bestimmte Strategien angewandt? Wenn ja, welche?*  [Did you use any sorting strategies? If yes, which ones?] | - | |
|  |  |  |  |  |  | x | *Haben Sie beim Ordnen der Sätze bestimmte Strategien angewandt? Wenn ja, welche?*  [Did you use any sorting strategies? If yes, which ones?] | - | |
|  |  |  |  | x |  |  | *Haben Sie beim Ordnen der Sätze in den ersten/letzten sechs Textabschnitten bestimmte Strategien angewandt? Wenn ja, welche?*  [Did you use any sorting strategies when arranging the sentences of the first/final six text passages? If yes, which ones?] | - | |
|  |  |  |  |  |  |  | **Test expected** | | |
|  |  | x |  |  | x |  | *Haben Sie mit einem anschließenden Lerntest gerechnet?*  [Did you expect the learning test?] | | |
|  |  |  |  |  |  |  | **Memory strategy use** | | |
|  |  |  |  |  |  | x | *Haben Sie Lernstrategien angewandt, um sich die Inhalte der Textabschnitte bis zum Lerntest zu merken? Haben Sie z.B. in der vergangenen Woche über die Inhalte nachgedacht, um die Informationen in Ihrem Gedächtnis zu behalten? Wenn ja, wie sind Sie dabei vorgegangen?*  [Did you use any learning strategies to memorize the texts’ content until the learning test? Have you for example thought about it in the past week, to memorize the information? If yes, how did you proceed?] | | |
|  |  |  |  |  |  |  | **Preparation** | | |
|  |  |  |  |  |  | x | *Bitte geben Sie wahrheitsgemäß an, ob Sie sich in der vergangenen Woche (willentlich oder unwillentlich) über die Studie hinaus zum Thema 'Lernen am Modell‘ informiert haben bzw. mit Informationen zum Thema konfrontiert wurden.*  [Please truthfully indicate whether you have, either intentionally or unintentionally, informed yourself about the topic ‘observational learning) beyond the scope of the study or have been confronted with information about the topic in the past week.] | | |

*Note.* ^a^Text comprehensibility was assessed separately after each text in Experiments 3 and 6 (incidental learning) as a cover task.

**Supplemental Material Table 2**

*GLMM Results for the Multiple-Choice Test Including Type of Question (Text-based vs. Inference-Based).*

|  | Exp. 3 | Exp. 5 | Exp. 6 | Exp. 7 |
| --- | --- | --- | --- | --- |
| Parameter | β (*SE*) | β (*SE*) | β (*SE*) | β (*SE*) |
|  | Fixed Effects | | | |
| Intercept | 1.29 (0.17)*** | 0.14 (0.27) | 1.95 (0.18)*** | 1.81 (0.15)*** |
| Learning condition^a^ | -0.03 (0.08) | -0.08 (0.04) | 0.17 (0.08)* | -0.08 (0.05) |
| Learning time^b^ | - | - | -0.11 (0.08) | - |
| Prior knowledge^a^ | - | - | 0.11 (0.06) | - |
| Test expected^c^ | 0.29 (0.13)* | - | -0.07 (0.12) | - |
| Generation/reading motivation^b^ | 0.07 (0.06) | -0.002 (0.06) | 0.02 (0.05) | - |
| Learning strategy use^a^ | -0.03 (0.08) | -0.02 (0.06) | 0.26 (0.09)** | - |
| Learning condition X Test expected | -0.14 (0.13) | - | -0.12 (0.12) | - |
| Learning Motivation^b^ | - | - | - | - |
| Test performance motivation^b^ | - | - | - | 0.02 (0.05) |
| Type of question^a^ | 0.01 (0.14) | -0.06 (0.26) | 0.23 (0.14) | 0.18 (0.15) |
| Learning condition X Type of question | -0.02 (0.04) | 0.06 (0.04) | 0.03 (0.02) | -0.04 (0.03) |
| Deviation score^b^ | - | -0.07 (0.14) | - | - |
| Coherence score^b^ | - | 0.06 (0.14) | - | - |
| Learning condition X deviation score | - | -0.07 (0.06) | - | - |
| Learning condition X coherence score | - | -0.05 (0.06) | - | - |
| Topic interest^b^ | - | - | 0.11 (0.05)* | - |
|  | Variance components | | | |
| Subjects | 0.19 (0.44) | 0.38 (0.62) | 0.21 (0.46) | 0.17 (0.41) |
| MC-option  Learning condition (RS) | 1.43 (1.20)  0.02 (0.15) | 5.18 (2.28) | 1.43 (1.20) | 1.69 (1.30)  0.01 (0.12) |

*Note*. ^a^contrast-coded, ^b^grand mean-centered, ^c^dummy-coded; learning condition: -1 = read, 1 = generate; prior knowledge: -1 = no, 1 = yes; test expected: 0 = no, 1 = yes; learning strategy use: -1 = no, 1 = yes; type of question: -1 = text-based, 1 = inference.

*p < 0.05, **p < 0.01, ***p < 0.001 (two-tailed)

**Supplemental Material Table 3**

*Multiple Choice Questionnaires–Version A.*

| Original questions in German | Correct |  | English Translation |
| --- | --- | --- | --- |
| 1. Laut Banduras sozialkognitiver Theorie sind Menschen in der Lage… |  |  | 1. According to Bandura’s social cognitive theory, people are capable of… |
| 1. ...neue Verhaltensweisen durch Imitation zu erwerben. | x |  | a) …acquiring new behaviors through imitation. |
| 1. ...vorauszusehen welche Konsequenzen ihr Verhalten mit sich bringen könnte. | x |  | b) …predicting what consequences their behavior might have. |
| 1. ...v.a. durch Ausprobieren neue Erkenntnisse zu erlangen. |  |  | c) …gaining new insights primarily through trial and error. |
| 1. ...sich nicht von anderen beeinflussen zu lassen. |  |  | d) …not being influenced by others. |
| 2. Welche der folgenden Aussagen ist/sind richtig? |  |  | 2. Which of the following statements is/are correct? |
| 1. Der Behaviorismus legt einen besonderen Schwerpunkt auf Gedanken und Absichten von Menschen. |  |  | a) Behaviorism places a particular focus on the thoughts and intentions of people. |
| 1. Bandura wird heute zu den Behavioristen gezählt. |  |  | b) Bandura is still classified as a behaviorist today. |
| 1. Wichtige Vertreter des Behaviorismus waren Skinner und Hull. | x |  | c) Important representatives of behaviorism were Skinner and Hull. |
| 1. Banduras erstes Werk wurde 1912 veröffentlicht. |  |  | d) Bandura’s first work was published in 1912. |
| 3. Welche der folgenden Aussagen zum Beobachtungslernen ist/sind richtig? |  |  | 3. Which of the following statements about observational learning is/are correct? |
| 1. Beobachtungslernen ist eine Lernform, die nur einen geringen Anteil unseres Lernens ausmacht. |  |  | a) Observational learning is a form of learning that accounts for only a small portion of our learning. |
| 1. Beobachtungslernen bezeichnet den Erwerb einer genauen Beobachtungsgabe im Verlauf der Entwicklung. |  |  | b) Observational learning refers to the acquisition of precise observational skills during development. |
| 1. Beobachtungslernen wurde auch im Tierreich gefunden. | x |  | c) Observational learning has also been found in the animal kingdom. |
| 1. Imitationslernen und Beobachtungslernen bezeichnen die gleiche Lernform. | x |  | d) Imitation learning and observational learning refer to the same form of learning. |
| 4. Bei welcher/welchen der folgenden Antworten kann es sich nach Banduras Theorie um ein Modell handeln? |  |  | 4. According to Bandura’s theory, which of the following could be considered a model? |
| 1. Eltern | x |  | a) Parents |
| 1. Comicfigur | x |  | b) Comic character |
| 1. Spielanleitung | x |  | c) Game instructions |
| 1. Lehrer | x |  | d) Teacher |
| 5. Welche der folgenden Aussagen zu Modellen ist/sind richtig? |  |  | 5. Which of the following statements about models is/are correct? |
| 1. Wenn Antons Fußballtrainer etwas vormacht und Anton ihn imitiert, spricht man von einem symbolischen Modell. |  |  | a) If Anton’s soccer coach demonstrates something and Anton imitates him, this is called a symbolic model. |
| 1. Ein Bild kann nicht als Modell dienen, weil es keine Instruktionen geben kann. |  |  | b) A picture cannot serve as a model because it cannot give instructions. |
| 1. Schauspieler fungieren selten als Modelle, weil meistens kein persönlicher Kontakt besteht. |  |  | c) Actors rarely serve as models because there is usually no personal contact. |
| 1. Eine wichtige Funktion von Modellen besteht darin, uns über die Konsequenzen unseres Verhaltens zu informieren. | x |  | d) An important function of models is to inform us about the consequences of our behavior. |
| 6. Welche der folgenden Aussagen ist/sind richtig? |  |  | 6. Which of the following statements is/are correct? |
| 1. Verstärkung bedeutet, dass ein Verhalten dadurch belohnt wird, dass es positive Folgen hat oder negative verhindert. | x |  | a) Reinforcement means that behavior is rewarded by positive outcomes or the prevention of negative ones. |
| 1. Bandura definiert vier Arten, auf die ein Verhalten verstärkt werden kann. |  |  | b) Bandura defines four ways in which behavior can be reinforced. |
| 1. Operantes Konditionieren ist eine bestimmte Form von Beobachtungslernen. |  |  | c) Operant conditioning is a specific form of observational learning. |
| 1. Verstärktes Imitationsverhalten tritt mit größerer Wahrscheinlichkeit erneut auf. | x |  | d) Reinforced imitation behavior is more likely to occur again. |
| 7. Auf welche der folgenden Arten kann laut Banduras Theorie ein Verhalten verstärkt werden? |  |  | 7. According to Bandura’s theory, in which of the following ways can behavior be reinforced? |
| 1. Direkt durch das Modell, dessen Verhalten imitiert wird. | x |  | a) Directly by the model whose behavior is being imitated. |
| 1. Durch die positive Konsequenz die das nachgeahmte Verhalten mit sich bringt. | x |  | b) Through the positive consequences of the imitated behavior. |
| 1. Dadurch, dass eine andere Person für dasselbe Verhalten verstärkt wird. | x |  | c) By seeing another person being reinforced for the same behavior. |
| 1. Dadurch, dass es dem Nachahmer gelingt, das Verhalten erfolgreich zu imitieren. |  |  | d) By the imitator successfully performing the behavior. |
| 8. Welche der folgenden Möglichkeiten ist ein Beispiel/ sind Beispiele für Verstärkung von imitiertem Verhalten? |  |  | 8. Which of the following examples is/are reinforcement(s) of imitated behavior? |
| 1. Anton wird von seiner Mutter gelobt, weil er den Tisch gedeckt hat, so wie er es bei seiner Schwester beobachtet hat. | x |  | a) Anton is praised by his mother for setting the table like he saw his sister do. |
| 1. Anton bekommt einen Nachtisch, weil er seinen Teller leer gegessen hat. |  |  | b) Anton gets dessert for finishing his plate. |
| 1. Antonia isst ihren Teller leer, weil sie gesehen hat, dass ihr Bruder Anton dafür einen Nachtisch bekommen hat. | x |  | c) Antonia finishes her plate because she saw her brother Anton get dessert for doing so. |
| 1. Anton muss, weil er heute Geburtstag hat, nicht beim Abwasch helfen. |  |  | d) Anton doesn’t have to help with the dishes because it’s his birthday. |
| 9. Welche der folgenden Komponenten sind zentral für das Beobachtungslernen? |  |  | 9. Which of the following components are central to observational learning? |
| 1. Aufmerksamkeit | x |  | a) Attention |
| 1. Kreativität |  |  | b) Creativity |
| 1. Motorische und physische Fähigkeiten | x |  | c) Motor and physical abilities |
| 1. Motivation | x |  | d) Motivation processes |
| 10. Im Matheunterricht erklärt der Lehrer wie man multipliziert. Anton sitzt aber nur gelangweilt auf seinem Platz und hört gar nicht richtig zu. An welchen/m Prozess/en liegt es wahrscheinlich, wenn das Beobachtungslernen bei Anton nicht erfolgreich ist? |  |  | 10. In math class, the teacher explains how to multiply. Anton, however, sits bored and doesn’t really listen. Which process(es) likely prevent observational learning from being successful in this case? |
| 1. Aufmerksamkeitsprozesse | x |  | a) Attention processes |
| 1. Behaltensprozesse |  |  | b) Retention processes |
| 1. Motorische Reproduktionsprozesse |  |  | c) Motor reproduction processes |
| 1. Motivationsprozesse | x |  | d) Motivation processes |
| 11. Im Sportunterricht erklärt die Lehrerin wie man einen Handstand macht. Antonia hat sich das Bein gebrochen und kann nicht mitmachen. An welchen/m Prozess/en liegt es wahrscheinlich, wenn das Beobachtungslernen bei Antonia nicht erfolgreich ist? |  |  | 11. In P.E. class, the teacher explains how to do a handstand. Antonia has a broken leg and can’t participate. Which process(es) likely prevent observational learning from being successful in this case? |
| 1. Aufmerksamkeitsprozesse |  |  | a) Attention processes |
| 1. Behaltensprozesse |  |  | b) Retention processes |
| 1. Motorische Reproduktionsprozesse | x |  | c) Motor reproduction processes |
| 1. Motivationsprozesse |  |  | d) Motivation processes |
| 12. Welche der folgenden Antwortmöglichkeiten ist/sind Bestandteile für eine erfolgreiche Nachahmung einer Turnübung? |  |  | 12. Which of the following answer options are components of successfully imitating a gymnastics exercise? |
| 1. Überwachung | x |  | a) Monitoring |
| 1. Korrektur | x |  | b) Correction |
| 1. Bewertung | x |  | c) Evaluation |
| 1. Regelmäßigkeit |  |  | d) Regularity |
| 13. Wovon hängt es u.a. ab, ob wir einem Modell unsere Aufmerksamkeit schenken? |  |  | 13. What does it depend on, among other things, whether we pay attention to a model? |
| 1. Verwandtschaftsgrad | x |  | a) Degree of relatedness |
| 1. Attraktivität | x |  | b) Attractiveness |
| 1. Einfluss | x |  | c) Influence |
| 1. Vertrautheit |  |  | d) Familiarity |
| 14. Nachdem eine neue Verhaltensweise angeeignet wurde, muss sie auch ausgeführt werden. Von welchen/welchem der folgenden Prozesse hängt dies ab? |  |  | 14. Once a new behavior has been acquired, it must also be executed. Which process(es) does this depend on? |
| 1. Aufmerksamkeit |  |  | a) Attention |
| 1. Belohnung | x |  | b) Reward |
| 1. Intelligenz |  |  | c) Intelligence |
| 1. Motivation | x |  | d) Motivation |
| 15. Anton beginnt mit 16 sehr viel Alkohol zu konsumieren. Welche/r der folgenden Gründe könnte/n laut Banduras Theorie dafür verantwortlich sein? |  |  | 15. Anton begins drinking a lot of alcohol at age 16. Which of the following reasons could be responsible according to Bandura’s theory? |
| 1. Anton hat gerade mit seiner Freundin Schluss gemacht. |  |  | a) Anton just broke up with his girlfriend. |
| 1. In Antons Lieblingsfilm geht es um einen coolen 16-jährigen, der auch Alkohol trinkt. | x |  | b) In Anton’s favorite movie, a cool 16-year-old also drinks alcohol. |
| 1. Anton mag einfach den Geschmack von Bier. |  |  | c) Anton simply likes the taste of beer. |
| 1. Seit seine große Schwester Antonia angefangen hat Alkohol zu trinken, hat sie viele neue Freunde. | x |  | d) Since his older sister Antonia started drinking, she has made many new friends. |
| 16. Welche/s der folgenden Beispiele beschreibt/beschreiben einen Modelleffekt? |  |  | 16. Which of the following examples describes a modeling effect? |
| 1. Der Tanzlehrer zeigt seinen Schüler(inne)n eine neue Tanzfigur, die diese dann nachmachen. | x |  | a) The dance teacher shows students a new dance move, and they imitate it. |
| 1. Antons Mutter schimpft, weil er seinen Teller nicht aufgegessen hatte. Das bringt seine Schwester Antonia dazu, schnell noch aufzuessen. |  |  | b) Anton’s mother scolds him for not finishing his food. This prompts his sister Antonia to finish hers quickly. |
| 1. Antonia gewinnt bei Ihrem Volleyballturnier und erntet dafür viel Anerkennung von ihren Eltern. Deshalb strengt sich Anton bei seinem Fußballspiel besonders an. |  |  | c) Antonia wins her volleyball tournament and receives much praise from her parents. As a result, Anton tries harder in his soccer game. |
| 1. In einem Film sieht Anton, wie ein Schauspieler etwas klaut. Als er das nächste Mal in einem Laden ist klaut Anton seit längerer Zeit auch mal wieder etwas. |  |  | d) In a film, Anton sees an actor steal something. The next time he’s in a store, he steals something again after a long time. |
| 17. Welche/s der folgenden Beispiele beschreibt/en einen Hemmungseffekt? |  |  | 17. Which of the following examples describes an inhibition effect? |
| 1. Anton erzählt Antonia, wie er bei seinem letzten Test geschummelt und trotzdem eine gute Note geschrieben hat. Beim nächsten Test schummelt Antonia ebenfalls. |  |  | a) Anton tells Antonia that he cheated on his last test and still got a good grade. Antonia cheats on the next test as well. |
| 1. Antons Mutter schimpft mit seiner Schwester, weil sie schon wieder viel zu spät nach Hause gekommen ist. Deshalb kommt Anton am nächsten Abend überpünktlich nach Hause. | x |  | b) Anton’s mother scolds his sister for coming home too late again. So Anton comes home extra early the next day. |
| 1. Anton lernt fleißig für einen Test und bekommt eine gute Note, deshalb lernt Antonia für ihren nächsten Test genauso fleißig. |  |  | c) Anton studies hard for a test and gets a good grade, so Antonia studies hard for her next test too. |
| 1. Anton muss nachsitzen, weil er im Unterricht gestört hat. Daraufhin ist seine ganze Klasse ruhig und arbeitet fleißig weiter. | x |  | d) Anton has detention for misbehaving in class. As a result, the entire class is quiet and works diligently. |
| 18. Welche/s der folgenden Beispiele beschreibt/en einen Enthemmungseffekt? |  |  | 18. Which of the following examples describe/s a disinhibition effect? |
| 1. Antonias Papa beginnt wieder mit dem Rauchen, weil er in einer Werbung jemanden genussvoll eine Zigarette rauchen sah. | x |  | a) Antonia’s dad starts smoking again after seeing someone enjoy a cigarette in an ad. |
| 1. Antonias neue beste Freundin meldet sich sehr häufig im Unterricht, Antonia beobachtet, wie sie sehr viel Lob dafür bekommt und meldet sich nun auch wieder häufiger. | x |  | b) Antonia’s new best friend participates frequently in class and receives a lot of praise, so Antonia also starts participating more. |
| 1. Antons Freund gewinnt einen Musikpreis, daraufhin beschließt Anton bei einem Malwettbewerb teilzunehmen. |  |  | c) Anton’s friend wins a music award, so Anton decides to enter a drawing competition. |
| 1. Ein Straftäter wird wieder rückfällig, nachdem er mitbekommt, wie sein Freund bei einem Überfall an viel Geld kommt. | x |  | d) A criminal reoffends after seeing his friend make a lot of money in a robbery. |
| 19. Welche der folgenden Aussagen ist/sind richtig? |  |  | 19. Which of the following statements is/are correct? |
| 1. Imitationslernen funktioniert auch im Mathematikunterricht. | x |  | a) Imitation learning also works in math class. |
| 1. Ein entfernter Verwandter hat als Modell meist mehr Einfluss als ein Elternteil. |  |  | b) A distant relative usually has more influence as a model than a parent. |
| 1. Wenn Schüler(innen) ein gänzlich neues Verhalten lernen, spricht man vom Enthemmungseffekt. |  |  | c) When students learn a completely new behavior, it is called a disinhibition effect. |
| 1. Wenn jemand ein Verhalten früher schon einmal gezeigt hat und es nun in Folge von Beobachtungslernen wieder zeigt, spricht man vom Modelleffekt. |  |  | d) When someone previously exhibited a behavior and now shows it again due to observational learning, it is called a modeling effect. |
| 20. Welche der folgenden Maßnahmen sind nach Banduras Theorie für den Schulalltag sinnvoll? |  |  | 20. Which of the following measures are sensible for everyday school life according to Bandura’s theory? |
| 1. Lehrer(innen) sollten auf ihr Verhalten achten, da sie als Vorbild dienen. | x |  | a) Teachers should be mindful of their behavior, as they serve as role models. |
| 1. Wenn Lehrer(innen) Schüler(innen) vor der ganzen Klasse zurechtweisen, wenn sie gestört haben, kann dies dafür sorgen, dass andere Schüler(innen) lernen, dass unerwünschtes Verhalten negative Konsequenzen hat. | x |  | b) If teachers scold students in front of the class for misbehaving, it may help others learn that undesirable behavior has negative consequences. |
| 1. Lehrer(innen) sollten ihren Unterricht an die motorischen und intellektuellen Fähigkeiten der Schüler(innen) anpassen, da diese sonst das Gelernte nicht umsetzten können. | x |  | c) Teachers should adapt their lessons to the motor and intellectual abilities of students, or they may be unable to apply what they learn. |
| 1. Lehrer(innen) sollten gutes Verhalten der Schüler(innen) eher nicht verstärken, da sie sonst zu übermütig werden. |  |  | d) Teachers should not reinforce good behavior in students, as this may make them overconfident. |

**Supplemental Material Table 4**

*Multiple Choice Questionnaires–Version B.*

| Original questions in German | Correct | Question Type |  | English Translation |
| --- | --- | --- | --- | --- |
| 1. Welche der folgenden Aussagen zu Bandura ist/sind zutreffend? |  | Text-based |  | 1. Which of the following statements about Bandura is/are correct? |
| 1. Bandura entwickelte zusammen mit Skinner die Social Cognitive Theory. |  |  |  | a) Bandura developed the Social Cognitive Theory together with Skinner. |
| 1. Albert Bandura wurde 1935 in Kanada geboren. |  |  |  | b) Albert Bandura was born in Canada in 1935. |
| 1. Bandura zählt zu den führenden Psychologen in der zweiten Hälfte des 20. Jahrhunderts. | x |  |  | c) Bandura is among the leading psychologists of the second half of the 20th century. |
| 1. Banduras frühe Theorien lassen sich ursprünglich dem Behaviorismus zuordnen. | x |  |  | d) Bandura's early theories can originally be assigned to behaviorism. |
| 2. Welche der folgenden Aussagen zu Banduras Theorie menschlichen Verhaltens ist/sind richtig? |  | Text-based |  | 2. Which of the following statements about Bandura’s theory of human behavior is/are correct? |
| 1. Motivationale Prozesse spielen bei Bandura eine geringe Rolle. |  |  |  | a) Motivational processes play a minor role in Bandura’s theory. |
| 1. Der Kognition wurde in Banduras Ansatz mehr Bedeutung zugemessen als im Behaviorismus. | x |  |  | b) Cognition is given more importance in Bandura’s approach than in behaviorism. |
| 1. In Banduras Ansatz ist die Antizipation von Verhaltenskonsequenzen bedeutsam. | x |  |  | c) Anticipation of behavioral consequences is important in Bandura’s approach. |
| 1. d) Soziale Aspekte werden bei Bandura vernachlässigt. (F) |  |  |  | d) Social aspects are neglected by Bandura. |
| 3. Welche der folgenden Aussagen zu den beim Beobachtungslernen auftretenden Prozessen trifft/treffen zu? |  | Text-based |  | 3. Which of the following statements about the processes involved in observational learning is/are correct? |
| 1. Fünf Prozesse sind nach Bandura am Beobachtungslernen beteiligt. |  |  |  | a) Five processes are involved in observational learning according to Bandura. |
| 1. Zu den Prozessen zählen auch Aufmerksamkeits- und Modelleffektprozesse. |  |  |  | b) The processes include attention and model-effect processes. |
| 1. Aufmerksamkeitsprozesse hängen vom Wert des Verhaltens und vom Wert des Modells ab. | x |  |  | c) Attention processes depend on the value of the behavior and the value of the model. |
| 1. Für Behaltensprozesse sind zwei Formen von Repräsentationen bedeutsam. | x |  |  | d) Two types of representations are important for retention processes. |
| 4. Welche(r) Effekt(e) beim Beobachtungslernen führt/führen nicht zum Lernen neuer Reaktionen? |  | Text-based |  | 4. Which effect(s) in observational learning do(es) **not** lead to learning new reactions? |
| 1. Der Modelleffekt |  |  |  | a) Model effect |
| 1. Der Enthemmungseffekt | x |  |  | b) Disinhibition effect |
| 1. Der Hemmungseffekt | x |  |  | c) Inhibition effect |
| 1. Der Auslöseeffekt |  |  |  | d) Elicitation effect |
| 5. Welche(s) der folgenden Szenarien ist/sind beispielhaft für den Auslöseeffekt? |  | Inference |  | 5. Which of the following scenarios exemplifies/exemplify the elicitation effect? |
| 1. Weil eine Schwester für ihre besonderen künstlerischen Fähigkeiten gelobt wird, ermutigt das ihre Geschwister, ihre künstlerischen Fähigkeiten ebenfalls zu schulen. |  |  |  | a) A sister is praised for her artistic talents, encouraging her siblings to develop their own. |
| 1. Angeregt durch seine Lieblingssängerin, die für ihre außergewöhnlichen Frisuren bekannt ist, färbt sich ein Fan die Haare pink. | x |  |  | b) A fan dyes their hair pink after being inspired by a favorite singer known for bold hairstyles. |
| 1. Weil ein beliebter Schüler erfolgreich im Orchester spielt, ermutigt dies einen Mitschüler, in die Theatergruppe einzusteigen. | x |  |  | c) A student joins the theater group after seeing a popular peer succeed in the orchestra. |
| 1. Weil ein beliebter Schüler erfolgreich im Chor singt, ahmen ihn seine Mitschüler nach und treten auch in den Chor ein. |  |  |  | d) A student joins the choir after observing a popular peer sing successfully in the choir. |
| 6. Andreas hat die letzte Prüfung mit einem Spickzettel geschrieben und seine Mitschüler Tobias und Ralf haben dieses Verhalten beobachtet. Tobias spickt selbst hin und wieder, wohingegen Ralf noch nie einen Spickzettel benutzt hat. Welche der folgenden Aussagen ist/sind korrekt? |  | Inference |  | 6. Andreas used a cheat sheet in the last exam, observed by Tobias and Ralf. Tobias occasionally cheats, but Ralf never has. Which of the following statements is/are correct? |
| 1. Wenn Andreas erwischt wird und durch die Prüfung fällt und Tobias deswegen beschließt in der Zukunft nicht mehr zu spicken, handelt es sich um einen Hemmungseffekt. | x |  |  | a) If Andreas gets caught and fails, and Tobias decides not to cheat anymore, it’s an inhibition effect. |
| 1. Wenn Andreas erwischt wird und durch die Prüfung fällt und dies bei Ralf die Reaktion auslöst, in der Zukunft auch nicht zu spicken, spricht man von einem Auslöseeffekt. |  |  |  | b) If Andreas gets caught and fails, and Ralf decides not to cheat, it’s an elicitation effect. |
| 1. Wenn Andreas nicht erwischt wird und die Note „Eins“ bekommt und dies Tobias veranlasst in der nächsten Prüfung ebenfalls zu spicken, handelt es sich um einen Enthemmungseffekt. | x |  |  | c) If Andreas is not caught and gets a top grade, and Tobias cheats next time, it’s a disinhibition effect. |
| 1. Wenn Andreas nicht erwischt wird und die Note „Eins“ bekommt und Ralf deswegen beschließt, die nächste Prüfung mit einem Spickzettel zu schreiben, handelt es sich um einen Modelleffekt. | x |  |  | d) If Andreas is not caught and gets a top grade, and Ralf decides to cheat next time, it’s a model effect. |
| 7. Die siebenjährige Paulina beobachtet die gleichaltrige Anna, wie sie im Pausenhof während der Schulpause ein Rad schlägt. Welche Aussage(n) trifft/treffen hier zu? |  | Inference |  | 7. Seven-year-old Paulina watches Anna, the same age, do a cartwheel in the schoolyard. Which statement(s) is/are correct? |
| 1. Anna ist kein Modell für Paulina, weil Anna genauso alt ist wie sie. |  |  |  | a) Anna is not a model for Paulina because they are the same age. |
| 1. Kinder lernen auch von anderen Kindern, aber nur, wenn sie das gleiche Geschlecht haben wie sie selbst. |  |  |  | b) Children only learn from other children of the same gender. |
| 1. Es gibt nicht nur menschliche Modelle beim Beobachtungslernen. | x |  |  | c) Models in observational learning are not limited to humans. |
| 1. Je mehr sich Paulina mit Anna identifizieren kann, desto wahrscheinlicher wird sie Annas Verhalten imitieren. | x |  |  | d) The more Paulina can identify with Anna, the more likely she is to imitate her behavior. |
| 8. Beim Turnen im Sportunterricht führt Maike eine komplizierte Kür am Schwebebalken vor, was die herumstehenden Kinder, einschließlich Theresa, beobachten. Welche der folgenden Aussagen trifft/treffen zu? |  | Inference |  | 8. During PE, Maike performs a difficult routine on the balance beam, observed by others including Theresa. Which statement(s) is/are correct? |
| 1. Wenn Theresa Maikes Kür nachmacht und dafür von Maike gelobt wird, verstärkt dies Theresas Imitationsverhalten. | x |  |  | a) If Maike praises Theresa for copying her, Theresa’s imitation behavior is reinforced. |
| 1. Wenn Theresa gut genug aufpasst, kann sie die Kür anschließend selbst fehlerfrei durchführen. |  |  |  | b) If Theresa pays close attention, she can perfectly perform the routine afterward. |
| 1. Wenn Maike von der Lehrerin gelobt wird, beeinflusst das zwar Maikes, aber nicht Theresas Nachahmungsverhalten. |  |  |  | c) Praise from the teacher affects Maike but not Theresa's imitation behavior. |
| 1. Die herumstehenden Kinder, die Theresa beobachten, hemmen sie, Maikes Kür nachzumachen. Deswegen spricht man hier vom Hemmungseffekt. |  |  |  | d) The observing children inhibit Theresa from copying Maike, which is an inhibition effect. |
| 9. Im Musikunterricht zeigt die Lehrerin den Schüler(inne)n wie man auf dem Xylophon eine bestimmte Melodie spielt. Was muss zutreffen, damit die Schüler(innen) die Melodie erfolgreich nachspielen können? |  | Inference |  | 9. In music class, the teacher shows students how to play a melody on the xylophone. What must be true for successful imitation? |
| 1. Die Schüler(innen) müssen aufmerksam zuschauen und hinhören. | x |  |  | a) Students must watch and listen attentively. |
| 1. Die Schüler(innen) müssen beim Üben darauf achten, wie ihre eigene Melodie klingt und sie mit der der Lehrerin vergleichen. | x |  |  | b) Students must compare their playing to the teacher’s while practicing. |
| 1. Die Befürchtung und Blamage, als einziges Kind die Melodie nicht nachspielen zu können, erhöht die Motivation der Schüler(innen), die Melodie nachzuspielen. | x |  |  | c) Fear of embarrassment for failing can increase motivation to imitate. |
| 1. Intellektuelle Fähigkeiten der Schüler(innen) beeinflussen ihr Imitationsverhalten nicht. |  |  |  | d) Intellectual abilities do not influence imitation behavior. |
| 10. Welche(s) der folgenden Szenarien ist/sind ein Beispiel für den Enthemmungseffekt? |  | Inference |  | 10. Which scenario(s) is/are examples of the disinhibition effect? |
| 1. Ein schneller Autofahrer mäßigt seine Fahrweise, da sein Bruder auf Grund von Geschwindigkeitsüberschreitungen seinen Führerschein abgeben musste. |  |  |  | a) A fast driver slows down after his brother loses his license due to speeding. |
| 1. Ein Schüler, der ab und an andere Mitschüler beschimpft, gibt sich zurückhaltend, nachdem er beobachtet hat, wie ein anderer Mitschüler wegen aggressiven Verhaltens einen Verweis bekommen hat. |  |  |  | b) A student who sometimes insults others becomes reserved after seeing someone else punished for aggression. |
| 1. Ein abstinenter Raucher fängt wieder mit dem Rauchen an, nachdem er eine Zigarettenwerbung gesehen hat, in der jemand genussvoll an einer Zigarette zieht. | x |  |  | c) A former smoker resumes smoking after seeing an ad of someone enjoying a cigarette. |
| 1. Ein ehemaliger Einbrecher wird rückfällig, nachdem ein Freund durch einen Einbruch an viel Geld gekommen ist. | x |  |  | d) A former burglar relapses after a friend makes a lot of money from a burglary. |
| 11. Im Sportunterricht macht der Lehrer den Schüler(inne)n einen Hocksprung über das Pferd vor, den sie heute lernen sollen. Welche der folgenden Aussagen ist/sind korrekt? |  | Inference |  | 11. In PE, the teacher demonstrates a squat jump over the vaulting horse. Which statement(s) is/are correct? |
| 1. Die guten motorischen Fähigkeiten von Schüler Chris unterstützen sein Beobachtungslernen. | x |  |  | a) Chris’s good motor skills support his observational learning. |
| 1. Eine verbale Repräsentation des Sprungs ist bei der Nachahmung besonders wichtig. |  |  |  | b) Verbal representation of the jump is especially important for imitation. |
| 1. Chris muss beim Nachahmen des Sprungs seine Ausführung selbst überwachen und ggf. korrigieren. | x |  |  | c) Chris must monitor and possibly correct his own execution of the jump. |
| 1. Der Enthemmungseffekt tritt hier auf, da Chris durch die Unterstützung seines Sportlehrers die Angst vor dem Sprung verliert. |  |  |  | d) The disinhibition effect occurs because Chris loses his fear due to the teacher's support. |
| 12. Welche(r) der folgenden Gründe könnte(n) schuld daran sein, wenn Modelllernen nicht stattfindet? |  | Text-based |  | 12. What reason(s) might prevent observational learning from occurring? |
| 1. Die beobachtende Person kann die beobachtete Person nicht leiden. | x |  |  | a) The observer dislikes the model. |
| 1. Es treten Defizite bei der sensorischen Repräsentation auf. |  |  |  | b) There are deficits in sensory representation. |
| 1. Das beobachtete Verhalten ist von geringer Relevanz für den Beobachter. | x |  |  | c) The observed behavior has little relevance to the observer. |
| 1. Das Modell wird für das gezeigte Verhalten belohnt, nicht aber die beobachtende Person. |  |  |  | d) The model is rewarded, but the observer is not. |
| 13. Welche der folgenden Aussagen trifft/treffen zu? Das „Bobo-Doll“- Experiment… |  | Text-based |  | 13. Which statement(s) about the "Bobo Doll" experiment is/are correct? |
| 1. …ist ein klassisches Beispiel für stellvertretende Verstärkung beim Modelllernen. |  |  |  | a) It is a classic example of vicarious reinforcement in model learning. |
| 1. …zeigte, dass Kinder aggressives Verhalten nachahmen, das zuvor jemand anderes gegenüber einer Puppe gezeigt hat. | x |  |  | b) It showed that children imitate aggressive behavior directed at a doll. |
| 1. …zeigte, dass Kinder nur verbale und keine physische Aggression nachahmten. |  |  |  | c) It showed that children only imitated verbal, not physical aggression. |
| 1. …löste bei allen Kindern aggressives Verhalten aus und ist daher v.a. ein empirischer Beleg für den Auslöseeffekt. |  |  |  | d) It triggered aggressive behavior in all children and is thus mainly empirical evidence for the elicitation effect. |
| 14. Welche Aussagen zum Modelllernen ist/sind richtig? |  | Text-based |  | 14. Which statement(s) about model learning is/are correct? |
| 1. Modelllernen beruht auf der klassischen Konditionierung. |  |  |  | a) Model learning is based on classical conditioning. |
| 1. Das zu imitierende Verhalten kann auf vier Arten verstärkt werden. |  |  |  | b) The behavior to be imitated can be reinforced in four ways. |
| 1. Die Folgen des nachgeahmten Verhaltens können eine verstärkende Wirkung haben. | x |  |  | c) The consequences of the imitated behavior can be reinforcing. |
| 1. Modelllernen hat viele Gemeinsamkeiten mit operanter Konditionierung. | x |  |  | d) Model learning shares many features with operant conditioning |
| 15. Welche der folgenden Aussagen ist/ sind zutreffend? Modelle... |  | Text-based |  | 15. Which statement(s) about models is/are correct? Models… |
| 1. …haben eine informative Funktion. | x |  |  | a) …have an informative function. |
| 1. …sind jegliche Repräsentationen eines Verhaltensmusters. | x |  |  | b) …are any representations of a behavior pattern. |
| 1. …können auch mündliche oder schriftliche Instruktionen sein. | x |  |  | c) …can be verbal or written instructions. |
| 1. …gibt es auch in der Tierwelt. | x |  |  | d) …exist in the animal world as well. |
| 16. Welche Form(en) der Verstärkung kommt/kommen beim Beobachtungslernen vor? |  | Text-based |  | 16. Which form(s) of reinforcement occur(s) in observational learning? |
| 1. Das Modell selbst wird für sein Verhalten belohnt. | x |  |  | a) The model is rewarded for its behavior. |
| 1. Das Modell selbst belohnt den Imitator. | x |  |  | b) The model rewards the imitator. |
| 1. Die unmittelbaren Folgen des nachgeahmten Verhaltens dienen als Belohnung. | x |  |  | c) The immediate consequences of the behavior act as reinforcement. |
| 1. Regelmäßiges Lob auch unabhängig vom gezeigten Verhalten verstärkt den Lerneffekt. |  |  |  | d) Regular praise regardless of behavior strengthens learning. |
| 17. Der zehnjährige Fabian beobachtet in seinem Lieblingsfilm im Fernsehen das aggressive Verhalten der Hauptfigur. Welche der folgenden Aussagen ist/sind korrekt? |  | Inference |  | 17. Ten-year-old Fabian watches his favorite movie where the main character behaves aggressively. Which statement(s) is/are correct? |
| 1. Die Konsequenzen, die die Hauptfigur aufgrund ihres aggressiven Verhaltens erfährt, können Fabians Nachahmungsverhalten beeinflussen. | x |  |  | a) The consequences the character experiences can influence Fabian’s imitation. |
| 1. Die Hauptfigur fungiert als symbolisches Modell. | x |  |  | b) The character serves as a symbolic model. |
| 1. Wenn Fabian aufgrund des Films aggressives Verhalten imitiert, das er zuvor noch nie gezeigt hatte, spricht man von einem Enthemmungseffekt. |  |  |  | c) If Fabian imitates previously unshown aggressive behavior, it’s a disinhibition effect. |
| 1. Durch das Beobachten der Hauptfigur kann Fabian entweder neue aggressive Verhaltensweisen erlernen oder es können bereits bei ihm vorhandene aggressive Verhaltensweisen ausgelöst bzw. gehemmt werden. | x |  |  | d) Fabian can either learn new aggressive behavior or trigger/inhibit existing ones. |
| 18. Welche der folgenden Aussagen bezogen auf die ablaufenden Prozesse beim Beobachtungslernen ist/sind korrekt? |  | Text-based |  | 18. Which statement(s) about the processes in observational learning is/are correct? |
| 1. Visuelle und verbale Repräsentationen sind immer gleich wichtig. |  |  |  | a) Visual and verbal representations are always equally important. |
| 1. Imitation erfordert die repräsentierten Handlungen in Verhalten umzusetzen. | x |  |  | b) Imitation requires translating represented actions into behavior. |
| 1. Motorische Reproduktionsprozesse sind bedeutsam für die Qualität der Imitation. | x |  |  | c) Motor reproduction processes are important for imitation quality. |
| 1. Motivation beeinflusst, ob beobachtetes Verhalten ausgeführt wird oder nicht. | x |  |  | d) Motivation affects whether observed behavior is executed. |
| 19. Welche der folgenden Aussagen trifft/treffen auf den schulischen Kontext zu? |  | Text-based |  | 19. Which statement(s) applies/apply in the school context? |
| 1. In der Schule spielt Beobachtungslernen keine Rolle. |  |  |  | a) Observational learning plays no role in school. |
| 1. Sowohl Mitschüler(innen) als auch Lehrer(innen) können als Modell fungieren. | x |  |  | b) Both peers and teachers can serve as models. |
| 1. Im Unterricht tritt der Modelleffekt, aber nicht der Hemmungseffekt auf. |  |  |  | c) Only the model effect, not the inhibition effect, occurs in class. |
| 1. Der Auslöseeffekt kommt in der Schule nicht vor. |  |  |  | d) The elicitation effect does not occur in school. |
| 20. Modelllernen tritt mit größerer Wahrscheinlichkeit auf, wenn… |  | Text-based |  | 20. Model learning is more likely to occur when… |
| 1. …negative Konsequenzen durch das Nachahmen einer Verhaltensweise verhindert werden können. | x |  |  | a) …negative consequences can be avoided through imitation. |
| 1. …positive Konsequenzen durch das Nachahmen einer Verhaltensweise zu erwarten sind. | x |  |  | b) …positive outcomes are expected through imitation. |
| 1. …das Modell vertrauenserweckend und einflussreich ist. | x |  |  | c) …the model appears trustworthy and influential. |
| 1. …das Modell ein Star ist. | x |  |  | d) …the model is a celebrity. |

**Supplemental Material Table 5**

*Multiple Choice Questionnaires–Version C.*

| Original questions in German | Correct | Question Type |  | English Translation |
| --- | --- | --- | --- | --- |
| 1. Welche der folgenden Aussagen zum Beobachtungslernen ist/sind richtig? |  | Text-based |  | 1. Which of the following statements about observational learning is/are correct? |
| 1. Beobachtungslernen ist eine Lernform, die nur einen geringen Anteil unseres Lernens ausmacht. |  |  |  | a) Observational learning is a form of learning that accounts for only a small portion of our learning. |
| 1. Beobachtungslernen bezeichnet den Erwerb einer genauen Beobachtungsgabe im Verlauf der Entwicklung. |  |  |  | b) Observational learning refers to the development of accurate observation skills over time. |
| 1. Beobachtungslernen wurde auch im Tierreich gefunden. | x |  |  | c) Observational learning has also been found in the animal kingdom. |
| 1. Imitationslernen und Beobachtungslernen bezeichnen die gleiche Lernform. | x |  |  | d) Imitation learning and observational learning refer to the same form of learning. |
| 2. Welche der folgenden Möglichkeiten ist ein Beispiel/ sind Beispiele für Verstärkung von imitiertem Verhalten? |  | Inference |  | 2. Which of the following is an/are example(s) of reinforcement of imitated behavior? |
| 1. Anton wird von seiner Mutter gelobt, weil er den Tisch gedeckt hat, so wie er es bei seiner Schwester beobachtet hat. | x |  |  | a) Anton is praised by his mother for setting the table, just like he saw his sister do. |
| 1. Anton bekommt einen Nachtisch, weil er seinen Teller leer gegessen hat. |  |  |  | b) Anton gets dessert because he ate everything on his plate. |
| 1. Antonia isst ihren Teller leer, weil sie gesehen hat, dass ihr Bruder Anton dafür einen Nachtisch bekommen hat. | x |  |  | c) Antonia eats all her food because she saw her brother Anton get dessert for doing the same. |
| 1. Anton muss, weil er heute Geburtstag hat, nicht beim Abwasch helfen. |  |  |  | d) Anton doesn’t have to help with the dishes because it's his birthday today. |
| 3. Welche der folgenden Komponenten sind zentral für das Beobachtungslernen? |  | Text-based |  | 3. Which of the following components is/are central to observational learning? |
| 1. Aufmerksamkeit | x |  |  | a) Attention |
| 1. Kreativität |  |  |  | b) Creativity |
| 1. Motorische und physische Fähigkeiten | x |  |  | c) Motor and physical abilities |
| 1. Motivation | x |  |  | d) Motivation |
| 4. Nachdem eine neue Verhaltensweise angeeignet wurde, muss sie auch ausgeführt werden. Von welchen/welchem der folgenden Prozesse hängt dies ab? |  | Text-based |  | 4. After a new behavior has been acquired, it must also be performed. Which of the following processes is/are responsible for this? |
| 1. Belohnung | x |  |  | a) Reward |
| 1. Motivation | x |  |  | b) Motivation |
| 1. Aufmerksamkeit |  |  |  | c) Attention |
| 1. Intelligenz |  |  |  | d) Intelligence |
| 5. Anton beginnt mit 16 sehr viel Alkohol zu konsumieren. Welche/r der folgenden Gründe könnte/n laut Banduras Theorie dafür verantwortlich sein? |  | Inference |  | 5. Anton starts drinking a lot of alcohol at the age of 16. Which of the following reasons could be responsible according to Bandura’s theory? |
| 1. Anton hat gerade mit seiner Freundin Schluss gemacht. |  |  |  | a) Anton just broke up with his girlfriend. |
| 1. In Antons Lieblingsfilm geht es um einen coolen 16-jährigen, der auch Alkohol trinkt. | x |  |  | b) Anton’s favorite movie features a cool 16-year-old who also drinks alcohol. |
| 1. Anton mag einfach den Geschmack von Bier. |  |  |  | c) Anton simply likes the taste of beer. |
| 1. Seit seine große Schwester Antonia angefangen hat Alkohol zu trinken, hat sie viele neue Freunde. | x |  |  | d) Since his older sister Antonia started drinking, she has made many new friends. |
| 6. Welche/s der folgenden Beispiele beschreibt/en einen Hemmungseffekt? |  | Inference |  | 6. Which of the following examples describe(s) an inhibition effect? |
| 1. Anton erzählt Antonia, wie er bei seinem letzten Test geschummelt und trotzdem eine gute Note geschrieben hat. Beim nächsten Test schummelt Antonia ebenfalls. |  |  |  | a) Anton tells Antonia how he cheated on his last test and still got a good grade. Antonia cheats on her next test as well. |
| 1. Antons Mutter schimpft mit seiner Schwester, weil sie schon wieder viel zu spät nach Hause gekommen ist. Deshalb kommt Anton am nächsten Abend überpünktlich nach Hause. | x |  |  | b) Anton’s mother scolds his sister for coming home too late again. As a result, Anton comes home very early the next evening. |
| 1. Anton lernt fleißig für einen Test und bekommt eine gute Note, deshalb lernt Antonia für ihren nächsten Test genauso fleißig. |  |  |  | c) Anton studies hard for a test and gets a good grade, so Antonia studies just as hard for her next test. |
| 1. Anton muss nachsitzen, weil er im Unterricht gestört hat. Daraufhin ist seine ganze Klasse ruhig und arbeitet fleißig weiter. | x |  |  | d) Anton has to stay after school for disrupting class. Consequently, the entire class stays quiet and works diligently. |
| 7. Welche/s der folgenden Beispiele beschreibt/en einen Enthemmungseffekt? |  | Inference |  | 7. Which of the following examples describe(s) a disinhibition effect? |
| 1. Antonias Papa beginnt wieder mit dem Rauchen, weil er in einer Werbung jemanden genussvoll eine Zigarette rauchen sah. | x |  |  | a) Antonia’s father starts smoking again after seeing someone enjoy a cigarette in a commercial. |
| 1. Antonias neue beste Freundin meldet sich sehr häufig im Unterricht, Antonia beobachtet, wie sie sehr viel Lob dafür bekommt und meldet sich nun auch wieder häufiger. | x |  |  | b) Antonia’s new best friend frequently participates in class; after seeing her receive a lot of praise, Antonia also starts participating more. |
| 1. Antons Freund gewinnt einen Musikpreis, daraufhin beschließt Anton bei einem Malwettbewerb teilzunehmen. |  |  |  | c) Anton’s friend wins a music award, so Anton decides to enter an art competition. |
| 1. Ein Straftäter wird wieder rückfällig, nachdem er mitbekommt, wie sein Freund bei einem Überfall an viel Geld kommt. | x |  |  | d) A criminal reoffends after seeing a friend make a lot of money in a robbery. |
| 8. Welche der folgenden Maßnahmen ist/sind nach Banduras Theorie für den Schulalltag sinnvoll? |  | Inference |  | 8. Which of the following measures is/are useful for everyday school life according to Bandura’s theory? |
| 1. Lehrer(innen) sollten auf ihr Verhalten achten, da sie als Vorbild fungieren. | x |  |  | a) Teachers should be aware of their behavior as they serve as role models. |
| 1. Wenn Lehrer(innen) Schüler(innen) vor der ganzen Klasse zurechtweisen, wenn sie gestört haben, kann dies dafür sorgen, dass andere Schüler(innen) lernen, dass unerwünschtes Verhalten negative Konsequenzen hat. | x |  |  | b) If teachers correct students in front of the whole class, it can teach others that undesirable behavior has consequences. |
| 1. Lehrer(innen) sollten ihren Unterricht an die motorischen und intellektuellen Fähigkeiten der Schüler(innen) anpassen, da diese sonst das Gelernte nicht umsetzten können. | x |  |  | c) Teachers should adapt their teaching to the motor and intellectual abilities of the students; otherwise, students may not be able to apply what they have learned. |
| 1. Lehrer(innen) sollten gutes Verhalten der Schüler(innen) eher nicht verstärken, da sie sonst zu übermütig werden. |  |  |  | d) Teachers should not reinforce good behavior too often, as students might become overconfident. |
| 9. Welche der folgenden Aussagen zu Bandura ist/sind zutreffend? |  | Text-based |  | 9. Which of the following statements about Bandura is/are correct? |
| 1. Bandura entwickelte zusammen mit Skinner die Social Cognitive Theory. |  |  |  | a) Bandura developed the Social Cognitive Theory together with Skinner. |
| 1. Albert Bandura wurde 1935 in Kanada geboren. |  |  |  | b) Albert Bandura was born in Canada in 1935. |
| 1. Bandura zählt zu den führenden Psychologen in der zweiten Hälfte des 20. Jahrhunderts. | x |  |  | c) Bandura is considered one of the leading psychologists of the second half of the 20th century. |
| 1. Banduras frühe Theorien lassen sich ursprünglich dem Behaviorismus zuordnen. | x |  |  | d) Bandura's early theories can originally be attributed to behaviorism. |
| 10. Welche der folgenden Aussagen zu den beim Beobachtungslernen auftretenden Prozessen trifft/treffen zu? |  | Text-based |  | 10. Which of the following statements about the processes involved in observational learning is/are correct? |
| 1. Fünf Prozesse sind nach Bandura am Beobachtungslernen beteiligt. |  |  |  | a) According to Bandura, five processes are involved in observational learning. |
| 1. Zu den Prozessen zählen auch Aufmerksamkeits- und Modelleffektprozesse. |  |  |  | b) The processes include attentional and modeling effect processes. |
| 1. Aufmerksamkeitsprozesse hängen vom Wert des Verhaltens und vom Wert des Modells ab. | x |  |  | c) Attentional processes depend on the value of the behavior and the value of the model. |
| 1. Für Behaltensprozesse sind zwei Formen von Repräsentationen bedeutsam. | x |  |  | d) Two types of representation are important for retention processes. |
| 11 Welche der folgenden Aussagen trifft/treffen zu? Das „Bobo-Doll“- Experiment… |  | Text-based |  | 11. Which of the following statements is/are correct? The “Bobo Doll” experiment… |
| 1. …ist ein klassisches Beispiel für stellvertretende Verstärkung beim Modelllernen. |  |  |  | a) …is a classic example of vicarious reinforcement in modeling. |
| 1. …zeigte, dass Kinder aggressives Verhalten nachahmen, das zuvor jemand anderes gegenüber einer Puppe gezeigt hat. | x |  |  | b) …showed that children imitate aggressive behavior previously demonstrated toward a doll. |
| 1. …zeigte, dass Kinder nur verbale und keine physische Aggression nachahmten. |  |  |  | c) …showed that children only imitated verbal aggression, not physical aggression. |
| 1. …löste bei allen Kindern aggressives Verhalten aus und ist daher v.a. ein empirischer Beleg für den Auslöseeffekt. |  |  |  | d) …triggered aggressive behavior in all children and is therefore primarily empirical evidence of the triggering effect. |
| 12. Welche der folgenden Aussagen ist/ sind zutreffend? Modelle... |  | Text-based |  | 12. Which of the following statements is/are correct? Models… |
| 1. …haben eine informative Funktion. | x |  |  | a) …have an informative function. |
| 1. …sind jegliche Repräsentationen eines Verhaltensmusters. | x |  |  | b) …are any representations of a behavior pattern. |
| 1. …können auch mündliche oder schriftliche Instruktionen sein. | x |  |  | c) …can also be oral or written instructions. |
| 1. …gibt es auch in der Tierwelt. | x |  |  | d) …also exist in the animal kingdom. |
| 13. Welche Form(en) der Verstärkung kommt/kommen beim Beobachtungslernen vor? |  | Text-based |  | 13. Which type(s) of reinforcement occur/s in observational learning? |
| 1. Das Modell selbst wird für sein Verhalten belohnt. | x |  |  | a) The model itself is rewarded for its behavior. |
| 1. Das Modell selbst belohnt den Imitator. | x |  |  | b) The model rewards the imitator. |
| 1. Die unmittelbaren Folgen des nachgeahmten Verhaltens dienen als Belohnung. | x |  |  | c) The immediate consequences of the imitated behavior serve as a reward. |
| 1. Regelmäßiges Lob auch unabhängig vom gezeigten Verhalten verstärkt den Lerneffekt. |  |  |  | d) Regular praise, even unrelated to behavior, enhances the learning effect. |
| 14. Welche der folgenden Aussagen trifft/treffen auf den schulischen Kontext zu? |  | Text-based |  | 14. Which of the following statements apply/applies to the school context? |
| 1. In der Schule spielt Beobachtungslernen keine Rolle. |  |  |  | a) Observational learning plays no role in school. |
| 1. Sowohl Mitschüler(innen) als auch Lehrer(innen) können als Modell fungieren. | x |  |  | b) Both classmates and teachers can serve as models. |
| 1. Im Unterricht tritt der Modelleffekt, aber nicht der Hemmungseffekt auf. |  |  |  | c) Only the modeling effect, but not the inhibition effect, occurs in class. |
| 1. Der Auslöseeffekt kommt in der Schule nicht vor. |  |  |  | d) The triggering effect does not occur in school. |
| 15. Modelllernen tritt mit größerer Wahrscheinlichkeit auf, wenn… |  | Text-based |  | 15. Modeling is more likely to occur when… |
| 1. …negative Konsequenzen durch das Nachahmen einer Verhaltensweise verhindert werden können. | x |  |  | a) …negative consequences can be avoided by imitating a behavior. |
| 1. …positive Konsequenzen durch das Nachahmen einer Verhaltensweise zu erwarten sind. | x |  |  | b) …positive consequences are expected from imitating a behavior. |
| 1. …das Modell vertrauenserweckend und einflussreich ist. | x |  |  | c) …the model is trustworthy and influential. |
| 1. …das Modell ein Star ist. | x |  |  | d) …the model is a celebrity. |
| 16. Andreas hat die letzte Prüfung mit einem Spickzettel geschrieben und seine Mitschüler Tobias und Ralf haben dieses Verhalten beobachtet. Tobias spickt selbst hin und wieder, wohingegen Ralf noch nie einen Spickzettel benutzt hat. Welche der folgenden Aussagen ist/sind korrekt? |  | Inference |  | 16. Andreas used a cheat sheet for the last exam, which was observed by his classmates Tobias and Ralf. Tobias cheats from time to time, while Ralf has never used a cheat sheet. Which of the following statements is/are correct? |
| 1. Wenn Andreas erwischt wird und durch die Prüfung fällt und Tobias deswegen beschließt in der Zukunft nicht mehr zu spicken, handelt es sich um einen Hemmungseffekt. | x |  |  | a) If Andreas is caught and fails the exam, and Tobias decides not to cheat in the future, this is an inhibition effect. |
| 1. Wenn Andreas erwischt wird und durch die Prüfung fällt und dies bei Ralf die Reaktion auslöst, in der Zukunft auch nicht zu spicken, spricht man von einem Auslöseeffekt. |  |  |  | b) If Andreas is caught and fails the exam, and this causes Ralf to also decide not to cheat in the future, this is called a triggering effect. |
| 1. Wenn Andreas nicht erwischt wird und die Note „Eins“ bekommt und dies Tobias veranlasst in der nächsten Prüfung ebenfalls zu spicken, handelt es sich um einen Enthemmungseffekt. | x |  |  | c) If Andreas is not caught and receives an A, and this causes Tobias to cheat on the next exam, this is a disinhibition effect. |
| 1. Wenn Andreas nicht erwischt wird und die Note „Eins“ bekommt und Ralf deswegen beschließt, die nächste Prüfung mit einem Spickzettel zu schreiben, handelt es sich um einen Modelleffekt. | x |  |  | d) If Andreas is not caught and gets an A, and Ralf decides to cheat on the next exam, this is a modeling effect. |
| 17. Beim Turnen im Sportunterricht führt Maike eine komplizierte Kür am Schwebebalken vor, was die herumstehenden Kinder, einschließlich Theresa, beobachten. Welche der folgenden Aussagen trifft/treffen zu? |  | Inference |  | 17. During gymnastics class, Maike performs a complicated routine on the balance beam while other children, including Theresa, watch. Which of the following statements is/are correct? |
| 1. Wenn Theresa Maikes Kür nachmacht und dafür von Maike gelobt wird, verstärkt dies Theresas Imitationsverhalten. | x |  |  | a) If Theresa imitates Maike’s routine and is praised by Maike, it reinforces her imitative behavior. |
| 1. Wenn Theresa gut genug aufpasst, kann sie die Kür anschließend selbst fehlerfrei durchführen. |  |  |  | b) If Theresa pays close enough attention, she can later perform the routine herself without mistakes. |
| 1. Wenn Maike von der Lehrerin gelobt wird, beeinflusst das zwar Maikes, aber nicht Theresas Nachahmungsverhalten. |  |  |  | c) If Maike is praised by the teacher, it influences Maike’s behavior but not Theresa’s imitative behavior. |
| 1. Die herumstehenden Kinder, die Theresa beobachten, hemmen sie, Maikes Kür nachzumachen. Deswegen spricht man hier vom Hemmungseffekt. |  |  |  | d) The children standing around watching Theresa inhibit her from copying Maike’s routine. This is called an inhibition effect. |
| 18. Welche(s) der folgenden Szenarien ist/sind ein Beispiel für den Enthemmungseffekt? |  | Inference |  | 18. Which of the following scenarios is/are an example of a disinhibition effect? |
| 1. Ein schneller Autofahrer mäßigt seine Fahrweise, da sein Bruder auf Grund von Geschwindigkeitsüberschreitungen seinen Führerschein abgeben musste. |  |  |  | a) A speeding driver slows down because his brother lost his license for speeding. |
| 1. Ein Schüler, der ab und an andere Mitschüler beschimpft, gibt sich zurückhaltend, nachdem er beobachtet hat, wie ein anderer Mitschüler wegen aggressiven Verhaltens einen Verweis bekommen hat. |  |  |  | b) A student who occasionally insults classmates becomes more reserved after seeing another student receive a warning for aggressive behavior. |
| 1. Ein abstinenter Raucher fängt wieder mit dem Rauchen an, nachdem er eine Zigarettenwerbung gesehen hat, in der jemand genussvoll an einer Zigarette zieht. | x |  |  | c) A former smoker starts smoking again after seeing a cigarette ad showing someone enjoying a cigarette. |
| 1. Ein ehemaliger Einbrecher wird rückfällig, nachdem ein Freund durch einen Einbruch an viel Geld gekommen ist. | x |  |  | d) A former burglar reoffends after a friend earns a lot of money through a burglary. |
| 19. Welche(r) der folgenden Gründe könnte(n) schuld daran sein, wenn Modelllernen nicht stattfindet? |  | Inference |  | 19. Which of the following reasons might explain why modeling does not occur? |
| 1. Die beobachtende Person kann die beobachtete Person nicht leiden. | x |  |  | a) The observer dislikes the person they are watching. |
| 1. Es treten Defizite bei der sensorischen Repräsentation auf. |  |  |  | b) There are deficits in sensory representation. |
| 1. Das beobachtete Verhalten ist von geringer Relevanz für den Beobachter. | x |  |  | c) The observed behavior is of little relevance to the observer. |
| 1. Das Modell wird für das gezeigte Verhalten belohnt, nicht aber die beobachtende Person. |  |  |  | d) The model is rewarded for the behavior, but the observer is not. |
| 20. Der zehnjährige Fabian beobachtet in seinem Lieblingsfilm im Fernsehen das aggressive Verhalten der Hauptfigur. Welche der folgenden Aussagen ist/sind korrekt? |  | Inference |  | 20. Ten-year-old Fabian watches his favorite film in which the main character behaves aggressively. Which of the following statements is/are correct? |
| 1. Die Konsequenzen, die die Hauptfigur aufgrund ihres aggressiven Verhaltens erfährt, können Fabians Nachahmungsverhalten beeinflussen. | x |  |  | a) The consequences experienced by the main character for their aggressive behavior can influence Fabian’s imitative behavior. |
| 1. Die Hauptfigur fungiert als symbolisches Modell. | x |  |  | b) The main character functions as a symbolic model. |
| 1. Wenn Fabian aufgrund des Films aggressives Verhalten imitiert, das er zuvor noch nie gezeigt hatte, spricht man von einem Enthemmungseffekt. |  |  |  | c) If Fabian imitates aggressive behavior he has never shown before due to the film, this is a disinhibition effect. |
| 1. Durch das Beobachten der Hauptfigur kann Fabian entweder neue aggressive Verhaltensweisen erlernen oder es können bereits bei ihm vorhandene aggressive Verhaltensweisen ausgelöst bzw. gehemmt werden. | x |  |  | d) By observing the main character, Fabian can either learn new aggressive behaviors or existing ones may be triggered or inhibited. |
